# Male mice song syntax depends on social contexts and influences female preferences

**DOI:** 10.3389/fnbeh.2015.00076

**Published:** 2015-04-01

**Authors:** Jonathan Chabout, Abhra Sarkar, David B. Dunson, Erich D. Jarvis

**Affiliations:** ^1^Department of Neurobiology, Duke University Medical CenterDurham, NC, USA; ^2^Howard Hughes Medical InstituteChevy Chase, MD, USA; ^3^Department of Statistical Science, Duke UniversityDurham, NC, USA

**Keywords:** song syntax, social contexts, Ultrasonic Vocalizations (USVs), playback, vocal communication

## Abstract

In 2005, Holy and Guo advanced the idea that male mice produce ultrasonic vocalizations (USV) with some features similar to courtship songs of songbirds. Since then, studies showed that male mice emit USV songs in different contexts (sexual and other) and possess a multisyllabic repertoire. Debate still exists for and against plasticity in their vocalizations. But the use of a multisyllabic repertoire can increase potential flexibility and information, in how elements are organized and recombined, namely syntax. In many bird species, modulating song syntax has ethological relevance for sexual behavior and mate preferences. In this study we exposed adult male mice to different social contexts and developed a new approach of analyzing their USVs based on songbird syntax analysis. We found that male mice modify their syntax, including specific sequences, length of sequence, repertoire composition, and spectral features, according to stimulus and social context. Males emit longer and simpler syllables and sequences when singing to females, but more complex syllables and sequences in response to fresh female urine. Playback experiments show that the females prefer the complex songs over the simpler ones. We propose the complex songs are to lure females in, whereas the directed simpler sequences are used for direct courtship. These results suggest that although mice have a much more limited ability of song modification, they could still be used as animal models for understanding some vocal communication features that songbirds are used for.

## Introduction

It has been long known that both wild and laboratory mice emit ultrasonic vocalizations (USVs) (Kalcounis-Rueppell et al., 2006), and possess a wide multisyllabic repertoire which can be divided, manually (Portfors, 2007; Scattoni et al., 2008) or automatically (Holy and Guo, 2005; Arriaga et al., 2012) in multiple categories based on spectral features, such as frequency modulation and/or duration. These USVs are emitted in various contexts, ranging from mother-pup retrieval behavior (D'Amato et al., 2005; Ehret, 2005), to juvenile interactions (Panksepp and Lahvis, 2007), opposite or same sex interactions (Moles et al., 2007; Chabout et al., 2012; Hanson and Hurley, 2012), and pain or other negative situations (Williams et al., 2008; Chabout et al., 2012). The USVs can be used as readouts of mouse models of neuropsychiatric, developmental or behavioral disorders (Bishop and Lahvis, 2011; Lahvis et al., 2011), and internal motivational states (Wohr and Schwarting, 2013). However, the role of adult USVs in different social contexts is just beginning to be investigated. Studies in wild mice suggested that some USVs are general territorial calls for neighbors (Petric and Kalcounis-Rueppell, 2013). Studies in laboratory mice suggest that USVs play an important role in social cohesion by triggering and maintaining the interaction between two individuals of the same sex (Chabout et al., 2012). Multiple studies have suggested that adult male mice also use their USVs for courtship, either to attract (or maintain) close proximity of females or facilitate actual mating (Pomerantz et al., 1983; White et al., 1998; Gourbal et al., 2004; Portfors, 2007; Hoffmann et al., 2009; Shepard and Liu, 2011; Sugimoto et al., 2011; Hanson and Hurley, 2012; Yang et al., 2013). These male USVs are thought, among other sensory cues, to convey reliable information on the emitter's condition that is potentially useful for female mate choice (Pasch et al., 2011; Asaba et al., 2014a).

In 2005, Holy and Guo (2005) advanced the idea that adult male mice USVs were organized as a succession of multisyllabic call elements or syllables similar to song of some songbirds. In other species, multisyllabic vocalizations has been proposed to can increase the potential flexibility and information carried by the songs, allowing the elements to be organized, combined and ordered in different ways, named syntax (different from syntax described for humans, which is known to also be associated with semantic meaning) (Berwick et al., 2011). Studies on song-learning in birds show that syntax changes are can be influenced by social context (Balaban, 1988; Byers and Kroodsma, 2009) and play a role in song structure and note use (Vignal et al., 2005; Hara et al., 2007; Byers and Kroodsma, 2009; Berwick et al., 2011). Variations in the syntax have been proposed to have an ethological relevance for sexual behavior and mate preferences, where usually more variable sequences are preferred (Jarvis, 2004a; Okanoya, 2004; Byers and Kroodsma, 2009). In mice, recent studies showed that adult males change the relative composition of syllable types they produce before, during, and after the presence of females (Hanson and Hurley, 2012; Yang et al., 2013). But so far, there is no evidence that mice alter their song syntax and possess distinct song types that are relevant for the females in ways comparable to songbird or mammal vocal learners. The evidence thus far suggest that mice have limited vocal plasticity (Grimsley et al., 2011; Kikusui et al., 2011; Arriaga et al., 2012; Hammerschmidt et al., 2012; Portfors and Perkel, 2014) and this is thought to be attributed to the absence or very sparse presence of a forebrain pathway that in vocal learners, like songbirds and humans, is well-developed (Fitch et al., 2010; Arriaga and Jarvis, 2013).

Here, while searching for the conditions that elicit the most robust and reliable USV songs from male mice, we found that male mice change their repertoire composition and use different syntax to build their songs when exposed to different social stimuli. We found that these differences in the songs are important for the listening female's preference. In the process, we also built computational and statistical approaches that allow for more quantitative analyses of mouse USV sequences and acoustic structure than in previous studies.

## Materials and methods

All experimental protocols were approved by the Duke University Institutional Animal Care and Use Committee (IACUC).

### Animals

Adult males and females of the B6D2F1/J strain were purchased from Jackson Laboratory (Bar Harbor, Maine). This strain has been previously used in our and other studies on mouse vocal communication studies (Holy and Guo, 2005; Kikusui et al., 2011; Arriaga et al., 2012). Before experiments, all mice were group housed (4–5 per cage) and kept on a 12-h light/dark cycle, and received *ad-libitum* food and water.

### Behavioral paradigm

**Figure 1:**
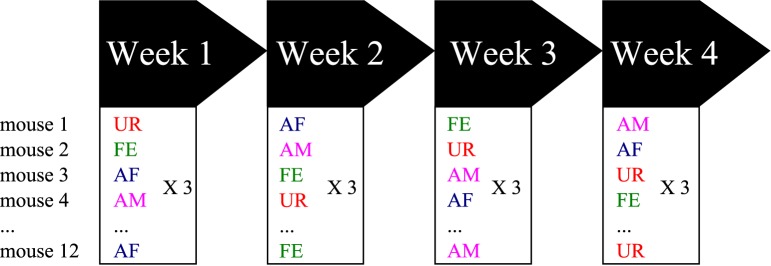
**Schematic representation of the behavioral singing paradigm**. Males were placed stimulated in one of four contexts each week, with different variables presented in a semi-random manner to prevent order effects. Abbreviations: UR, Fresh female urine; FE, alive female; AF, anesthetized female; AM, anesthetized male.

At 8 weeks old (young adults), 12 males were sexually socialized by spending one night with a sexually mature female (different than the females used for playback). Previous work showed that prior exposure of a male mouse to a sexually mature female several or more days before conducting the experiments increased the male's motivational state to exhibit courtship USV behavior when tested (Arriaga et al., 2012). After the overnight experience, the male mice were placed back in same sex social housing (4 males per cage) until the test day. The males were then removed from their cages, placed in a new cage and then singly habituated in the sound recording environment (15″ × 24″ × 12″ beach cooler with a tube for pumped air circulation input, no light and a hanging microphone, as a soundproof compartment) for 15 min, where 5 min of control recordings were made during the habituation period. Then, we exposed the males to one of the following different stimuli from animals the males has not had experience with: (1) fresh urine collected from at least two different females (UR) or males (URM) from two distinct cages (and mixed) within minutes of exposure on a urine-dipped cotton tip placed inside the male's cage; (2) awake and behaving adult female (FE); (3) an anesthetized adult female (AF); and (4) an anesthetized adult male (AM). The experimental stimulus (urine or another animal) was placed inside the subject mouse's cage or on the metal lid of the cage (anesthetized animal) for 5 min. Thus, the same mouse was exposed on three consecutive days to the same stimulus category (either AM, AF, FE or urine), but the identity of the stimulus (specific animal) was changed every day (over a 3 days period) to ensure against a familiarity effect (Figure 1). Then the next week, the same mouse was exposed to a different stimulus category following the same procedure. We repeated this for 4 consecutive weeks, where order of stimulus presentation was shuffled between weeks such that each animal received a different stimulus order set to normalize against any possible order effect. We tried to use females in pro-estrus or estrus (wide vaginal opening and pink surround) for the female stimuli when possible with the timed schedule of randomized conditions. The AF and AM animals were anesthetized with ketamine/xylazine (100 and 10 mg/kg, respectively, i.p.) and put on a heating pad outside of the test cage between recording sessions for at least 5 min. Between trials, the mouse cage was cleaned with 1% Trifectant and water. We did not do awake male recordings as the males can be very aggressive with each other, fighting instead of producing USV songs and when they do vocalize it is under specific motivational conditions where there is no resident/intruder hierarchy (Chabout et al., 2012).

Sounds were recorded with UltraSoundGate CM16/CMPA ultrasound microphones suspended over the center of each cage in the recording box, high enough so that the receiving angle of the microphone covered the whole area of the test cage. The microphones were connected to a multichannel ultrasound recording interface Ultrasound Gate 416H, which was itself plugged into a computer equipped with Avisoft Recorder USG software v4.2.18 (Sampling frequency: 250 kHz; FFT-length: 1024 points; 16-bits). All recording hardware and software were from Avisoft Bioacoustics® (Berlin, Germany).

All the recording files are available on the mouseTube platform (https://mousetube.pasteur.fr).

### Acoustic definitions

Following our standard definitions (Arriaga and Jarvis, 2013), a sound note is the most basic acoustic unit, and is formed by a single continuous sound with or without variations in fundamental frequency. One or more notes can be combined to form a “call” or “syllable,” which are reproducible single acoustic units separated by periods of silence. We distinguish “syllables” from “calls” by the pattern of usage. Calls are typically produced in isolation or in short burst and may obtain semantic content on their own. Syllables, however, derive their classification from being included in a larger unit representing a longer series of rapidly produced vocalizations of varying types. A song is a sequence of vocalizations, often elaborate, delivered periodically and sometimes with rhythm.

### Sound analysis

Acoustic waveforms were processed using a custom MATLAB program (Arriaga et al., 2012), originally modified from code written by Timothy E. Holy (Holy and Guo, 2005) that we call “Mouse Song Analyzer v1.3” and available on our website (http://jarvislab.net/research/mouse-vocal-communication/). Briefly, the software computed the sonograms from each waveform (256 samples/block, half overlap), thresholded to eliminate the white noise component of the signal, and truncated for frequencies outside the USV song range (35–125 kHz). We used a criterion of 10 ms minimum to separate two syllables and 3 ms as the minimum duration of a syllable. The identified syllables were then classified by presence or absence of instantaneous “pitch jumps” separating notes within a syllable into four categories: (1) simple syllable without any pitch jumps (type “s”); (2) complex syllables containing two notes separated by a single upward (“u”) or (3) downward (“d”) pitch jump; and (4) more complex syllables containing a series of multiple pitch jumps (type “m”). Any sounds the software could not classify were put into “Unclassified” category and made up, respectively 2.3% of the repertoire in UR, 5% in FE, 2.6% in AF, 16% in AM. Manual visual inspection of the sonograms of the unclassified sounds revealed that most of them were either syllables that overlapped with mechanical, non-vocal noises the mouse made, such as scratching, walking on the plastic cage, and chewing on the cage lid, or non-vocal mechanical sounds that included frequencies that reached above our 25 kHz cut off (Figure S1). Some were human audible non-USV calls that had harmonics in the USV range, and others were very rarely produced highly modulated complex syllables, particularly in the UR condition (Figure S1). The non-vocal sounds made up the vast majority of examined unclassified sounds in the AM condition, which we believe is due to more physical aggressive interactions with the anesthetized male and this is why it has the highest percentage of this category. Comparisons between automated and manual methods on example sonograms from all syllable categories lead to about 95% overlap.

**Figure 2:**
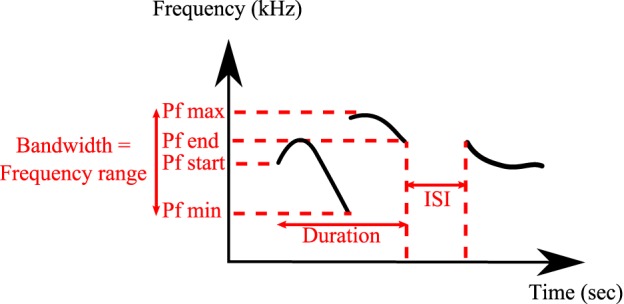
**Detailed spectral features calculated by MATLAB® Software**. Duration, inter-syllable interval (ISI), peak frequency min (Pf min), peak frequency max (Pf max), peak frequency start (Pf start), peak frequency end (Pf end), and bandwidth.

All analyses were conducted on a total of 24,320 classified syllables in UR, 29 in URM, 16,217 in AF, 22,184 in FE and 2743 in AM from 12 males. The following spectral features were calculated automatically by the MATLAB code from the sonograms of each of the classified syllables types: Syllable duration, inter-syllable interval, standard deviation of pitch distribution, pitch (mean frequency), frequency modulation, spectral purity, and bandwidth (Figure 2). Spectral purity was calculated as the instantaneous maximum power at the peak frequency normalized by the instantaneous total power in the spectrum, averaged across the entire syllable; a pure tone has a spectral purity of 1, and white noise approaches 0.

### Syntax analysis using probabilities

We performed two types of syntax probability analyses: one general and one conditional. For both analyses, we generated a custom script in Microsoft Excel (2003) that detects silences (gap >250 ms), and letter-coded sequences of syllables and silence (e.g., s-d-u-m-s-Silence…). Then, for the *general probability analyses*, we used an approach modified from Ferreira et al. (2006) to estimate the overall probability of occurrence of each transition type as the ratio of the total of number transitions of that type to the total number of transitions of all types:

$$Probability\left(\begin{array}{c} occurence\;of\;a\\ transition\;type \end{array}\right)\;=\frac{\begin{array}{c} total\;number\;of\;occurrences\;\\ of\;a\;transition\;type \end{array}}{\begin{array}{l} Total\;number\;of\;transition\;\\ \;of\;all\;types \end{array}}$$

*For the conditional analyses*, to get an idea of relative syllable transition dynamics, we estimated conditional probabilities of different transition types given the starting syllable of the transition:

$$\begin{array}{l} Probability\left(\begin{array}{c} occurence\;of\;a\;transition\;type\\ given\;the\;starting\;syllable \end{array}\right)\\ \;\;\;\;\;\;\;\;=\;\frac{\begin{array}{c} Total\;number\;of\;occurences\;of\;a\\ \;transition\;type \end{array}}{\begin{array}{c} Total\;number\;of\;occurences\;of\;all\\ \;transition\;types\;with\;the\;same\;starting\\ syllable \end{array}} \end{array}$$

First to assess whether the syllable generation mechanism is random, we tested whether a previous syllable was predictive of the next syllable in a sequence. In particular, we tested the null hypothesis of whether there was proportional probability for the generation of a syllable across all preceding syllables and/or silence. The null hypothesis is that the probability of a mouse using syllable *k* under context *c* does not depend on the previously used syllable *j* but is instead generated randomly according to the mouse's overall preference for *k* in context *c*. Second, we investigated whether the transition mechanisms vary significantly across different contexts. For a simple alternative hypothesis that is powerful to a broad class of sequential dependence structures, we assume a Markov model for the syllable transitions. Pearson's chi-squared tests were used along the lines of Xie and Zimmerman (2014) as described in the Supplementary Material (Appendix A). Both the Excel-based sequence analysis and the R code used for the statistical comparisons are available on our website (http://jarvislab.net/research/mouse-vocal-communication/) and in the Supplementary Material.

From both the general and conditional probabilities, we created syntax diagrams in Graphviz v2.36 (http://www.graphviz.org), with nodes designating different syllable categories and with arrow color and/or thickness pixel size proportional to ranges of probability values between syllables. For clarity, for the general probability, we only represent transitions higher than 0.005 (higher than 0.5% of chance occurrence). For the conditional probabilities, we used a threshold of 0.05 because each probability in the “overall model” is lower considering that we divide by the total number of syllables and not only by one specific type.

### Playback behavioral experiment

B6D2F1 females (*n* = 10, 7–15 weeks old) for the playback experiment were exposed to a male for 3 days before the experiment to trigger estrus, using the Whitten effect (Whitten, 1956). The female mice were housed in a cage with a barrier, which separated the male and the female, to prevent physical mating, but still allow social contact and scent smelling. We visually followed the estrus cycle and when pro-estrus or estrus was evident (vaginal opening and pink surround), females were put back together in a social housing cage until the playback experiment on the next day. The females were then habituated singly for 10 min in a Y-maze with opaque arms of 30 cm, closed to access to the other arms by a piece of Plexiglass®. Away from the “starting” arm, were two speakers (Polaroid, Avisoft Bioacoustic, Berlin, Germany) placed at the extremity of two arms of the Y through a round hole, at floor level of the arm. After the 10 min habituation period, one speaker on one side played a male song previously recorded during the UR context and the other speaker simultaneously played a song from of the same male from the FE context, for 5 min. The complex and simple songs for each exemplar are from up to three song bouts stitched together, representative of songs from a single male. We made each stimulus ~15 s long, to prevent amount of song heard being a variable. We used pairs of songs from three males, each containing, respectively 85, 89, 92 syllables for the complex and 65, 92, 92 syllables for the simple songs, to make sure if a preference was found, it would not be because of a bias to one song of one male.

The speakers were connected to an UltraSoundGate Player 216H (Avisoft Bioacoustic), using Avisoft Recorder USGH version 4.2.18 and had a frequency range (±12 dB as the maximum deviation from the average sound volume) of 25–125 kHz. We adjusted the loudness between the channels by controlling the level of the peak power directly on the playback software (Avisoft Recorder plugged to Ultrasound gate 216H) before the experiment and we matched, when necessary, peak powers between left and right using the software's knobs (little white bars), to prevent sound volume from being a variable. The recordings are from the same experimental males used in the context analyses, and the playbacks possess the same fundamental SPL (average SPL was centered at −60 dB). Using two microphones, we also made sure that both songs were audible at the entrance of both choice arms so the female can choose.

Each pair of male songs was played from each speaker using the loop mode of Avisoft Recorder for 5 min non-stop. During the 5-min period, the female was allowed in the maze and the time she spent in each arm was counted. After one session, the female was placed back in the starting arm and closed again by a piece of Plexiglass® for 1 min before the next session, and this was repeated for a total of 4 sessions × 5 min. To avoid measuring a possible side bias during the test (the females choosing always the same side regardless of the song played), we switched sides for the playback signal each time between sessions. The starting side for a given song type on the very first session was random, so that there would not be any switching order effect found. This entire experiment was repeated three times for three exemplar songs at three different weeks. The maze was cleaned between sessions and between females with 70% alcohol and distilled water, and allowed to fully dry before the next session or female.

### Statistical analyses

Statistical analyses on repertoire composition and acoustic features were conducted using either with IBM SPSS Statistic software (v.22.0) or R (R Development Core Team, 2011). Repeated measures ANOVA or MANOVA were used to compare male subject performances across stimuli. For the repeated measures ANOVA, when the assumption of sphericity was violated (Mauchly's test) we report the corrected degrees of freedom using Greenhouse-Geisser correction. *Post-hoc* analyses were performed using Student's paired *t*-test comparisons (for dependent variables) when appropriate. We controlled the false discovery rate due to multiple comparisons by using Benjamini and Hochberg (1995) correction as it was appropriate for our sample sizes, and the threshold was adjusted accordingly and detailed in the figure captions and tables.

## Results

### Syllable rate and repertoire composition depend on the social context

Our experiments were motivated by a need to find conditions that would elicit the most robust amount (number of syllables/min) and reliable (percentage of animals) singing behavior from AM mice, because obtaining sufficient amount of song behavior for analyses had not been reliable in our hands in previous studies (Arriaga et al., 2012; Chabout et al., 2012). We tried to mimic and control different situations the mice could encounter in the wild. Once the conditions were found, we then discovered social context differences. First, we describe the results on singing amount from the various conditions.

**Figure 3:**
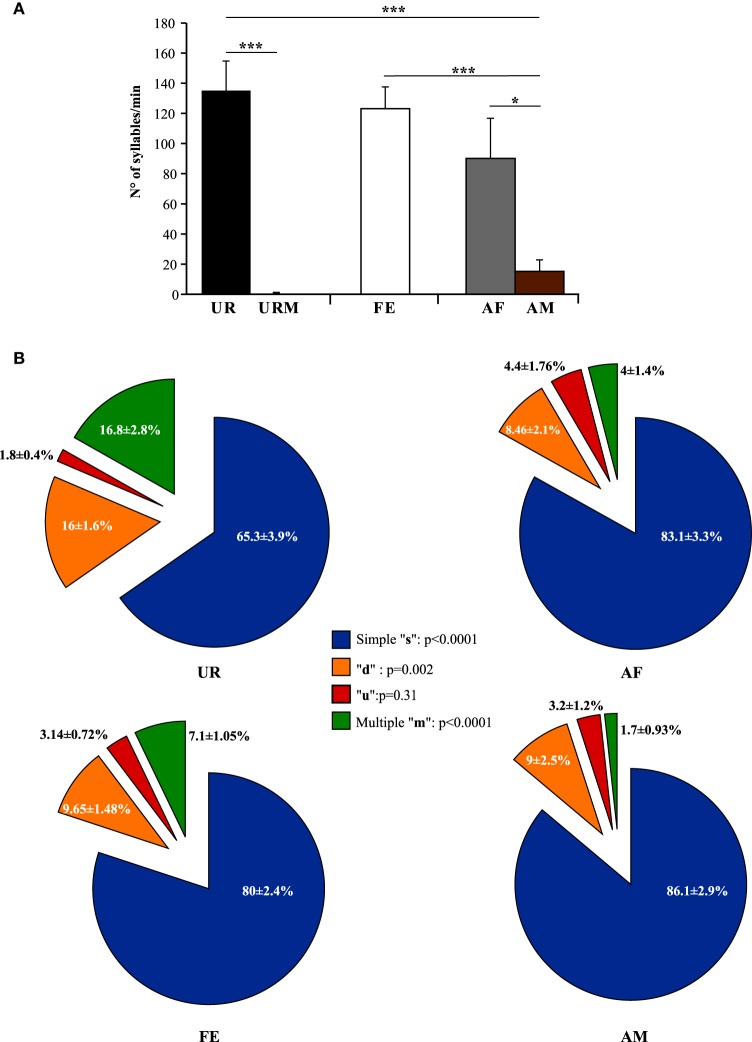
**Number of syllables emitted and repertoire across conditions**. **(A)** Call rate (number of USVs per minute). Data are presented as mean ± SEM (*n* = 12 males). *p < 0.03, ***p < 0.0001 for *post-hoc* Student's paired *t*-test after Benjamini and Hochberg correction. (**B**) Repertoire compositions in urine (UR), anesthetized female (AF), live female (FE), anesthetized male (AM) conditions. Central statistics refer to the repeated measure ANOVA across contexts for a given syllable.

Generally, our anecdotal observations was consistent with previously-reported differences in eliciting greater amount of song from males with freshly collected overnight female urine than frozen (Hoffmann et al., 2009), but we found that fresh female urine (UR) collected within 2 min of presentation to a male elicited the highest number of syllables and reliable singing from all (*n* = 12) males (Figure 3A; ~130 syllables/min). By contrast, when males were presented with fresh male urine (URM) collected within 2 min of presentation, there was very little to no singing, depending on animal (Figure 3A; total of 29 syllables for 12 males). This was similar to the very few or no syllables (depending on animal) the males produced during the habituation phase with no stimulus (total of 26 syllables for 12 males), showing that the URM does not elicit more USV beyond the normal background vocalization rate under our conditions. In comparison, all male mice (*n* = 12) sang in response to a live sexually experienced female (FE) and at comparable levels with fresh female urine (Figure 3A). Males still sang considerable amounts, although some less, with an anesthetized female (AF; Figure 3A; Table S1A), demonstrating that reciprocal social behavior was not necessary for them to sing robust amounts of song. In the FE condition, we cannot exclude that the female was vocalizing, but previous studies (Whitney et al., 1973) showed that in a pair of one female and one male, ultrasonic vocalizations were often detected when only the male was awake and never detected when only the female was awake. Consistent with this, we found no difference in the number of syllables emitted in both FE and AF contexts, where the only difference is the awake female. If the females were producing a lot of USVs, we would see a strong difference between these two conditions. In addition, we have never observed females to sing long sequences of syllables under any conditions tested (not shown and see Moles et al., 2007; Von Merten et al., 2014). Finally, these same males sang short sequences and fewer syllables overall in the presence of an anesthetized male (AM; Figure 3A; Table S1A), indicating that it is not simply the presence of another animal that elicited robust singing, but the presence of female stimuli. Overall, very fresh female urine and awake or anesthetized females represent the more salient stimuli for all males tested.

**Figure 4:**
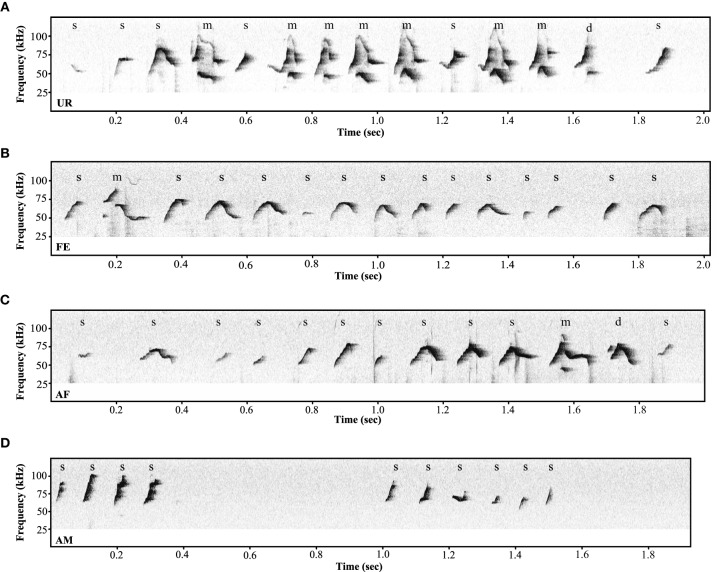
**Variety of ultrasonic songs examples in male mice recorded in different conditions**. **(A)** urine (UR), **(B)** live female (FE), **(C)** anesthetized female (AF), **(D)** anesthetized male (AM) conditions. “s” represents the simple syllables without any frequency jumps, “d” and “u” represent syllables with only one frequency jump, and “m” syllables with multiple frequency jumps.

Under the conditions that elicited sufficient syllables from enough animals to perform statistical analyses (more than 10 syllables per individual per condition), we noted qualitative and quantitative differences in repertoire composition between social contexts. In all contexts, male mice produced the simpler syllable type without pitch jumps, “s,” more often than all other types (Figure 3B). However, in the presence of fresh female urine they produced significantly less “s” type, and more down “d” and multiple “m” pitch jump types (Figure 3B; Table S1B). The relative proportion of the up “u” pitch jump syllable was similar across contexts. Interestingly, the low standard error of the mean of repertoire composition for each syllable type among animals for each context demonstrates that the differences among contexts are very similar for each animal. These quantitative song differences in context were qualitatively seen in sonograms of songs of 1 s or longer (Figure 4). We interpret these findings to mean that males produce complex syllables more often in response to female urine than in response to live animals.

### Males sing syllables at louder, longer, and higher pitch in response to fresh female urine, but sharper to awake females

**Figure 5:**
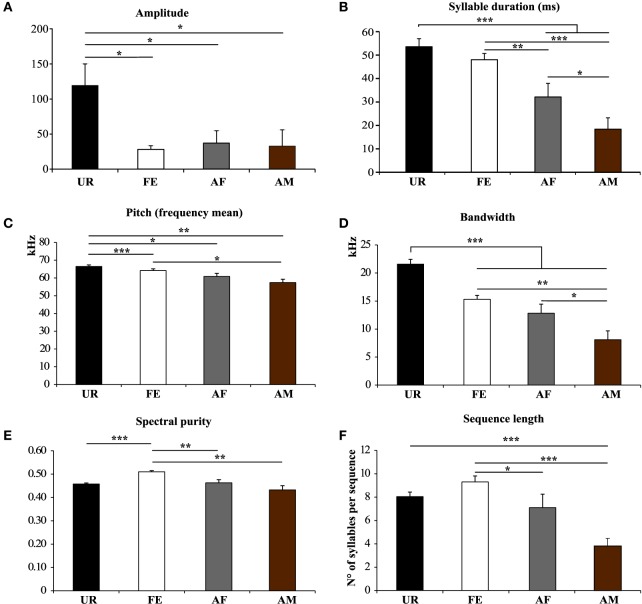
**Characteristics of syllables and sequence length in different contexts. (A)** Amplitude. *p < 0.025 after Benjamini and Hochberg correction. **(B)** Syllable Duration (milliseconds) *p < 0.041, **p < 0.005, ***p < 0.0001 after correction. **(C)** Frequency mean. *p < 0.033, **p < 0.005, ***p < 0.0001 after correction. **(D)** Frequency range or Bandwidth. *p < 0.041, **p < 0.005, ***p < 0.0001 after correction. **(E)** Spectral purity of the syllables. **p < 0.005, ***p < 0.0001 after correction. **(F)** Length of the sequences in number of syllables per sequence. *p < 0.025, ***p < 0.0001 after correction. Data are presented mice as mean ± SEM (*n* = 12 males). Abbreviations: urine (UR), anesthetized female (AF), live female (FE), anesthetized male (AM).

**Figure 6:**
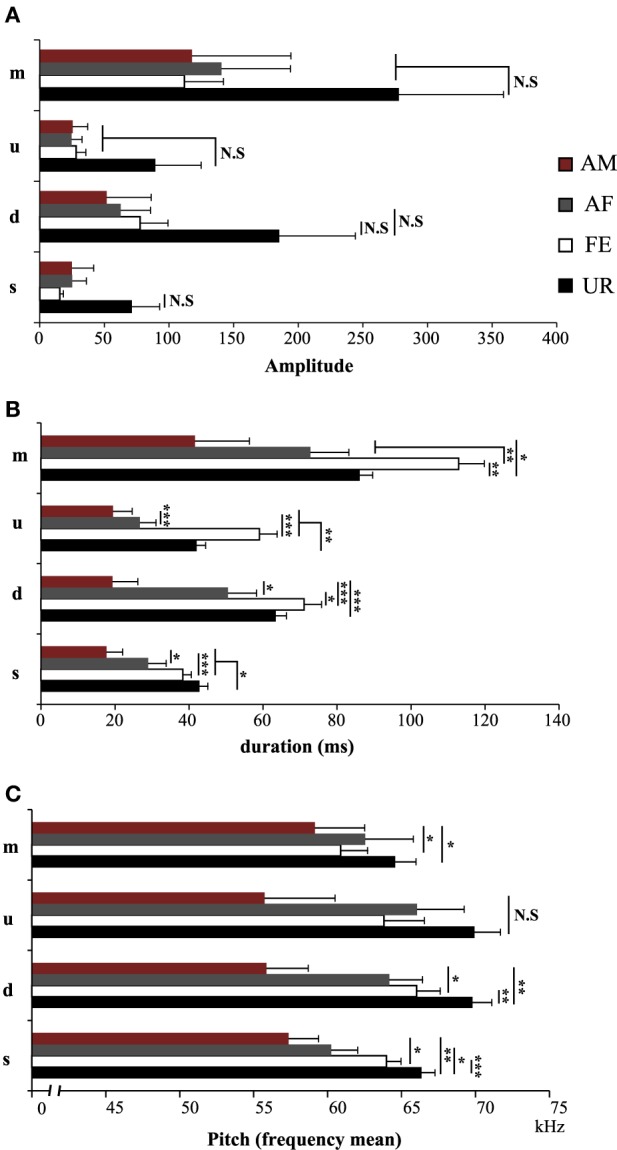
**Characteristics of individual syllables in different contexts**. **(A)** Amplitude of individual syllable types. NS, Non significant. **(B)** Duration of individual syllable types. **(C)** Pitch (frequency mean) of individual syllable types. Data are presented as mean ± SEM (*n* = 12 males). *p < 0.05, **p < 0.005, ***p < 0.0001 for *post-hoc* Student's paired *t*-test after Benjamini and Hochberg correction. NS, Non significant.

We also noticed that the males produced syllables up to four times louder in the urine condition than in all the other conditions (Figure 5A; Table S2A). Analyzing syllables individually, all had louder means in the urine context, and “s” and “d” statistically so, but after Benjamini and Hochberg correction these differences were not significant (Figure 6A; Table S3A). We interpret this difference in analyses to indicate that there is a high degree of variance in the loudness, and that the significant difference in loudness is seen with a larger sample size of all syllables combined. Males produced their syllables with longer duration and higher in the urine and awake female context relative to other context, with the shortest duration in the anesthetized male context (Figure 5B; Table S2B). The differences in duration were mainly due to longer “s” type syllables in the UR context and the three other types of syllable syllables in the awake female context (Figure 6B; Table S3B). Pitch (frequency mean) followed a similar pattern (Figure 5C; Table S2C), although this trend existed comparably for all syllable types (Figure 6C; Table S3C). Syllable bandwidth also followed the same pattern (Figure 5D; Table S2D), consistent with the males singing more complex syllables in the urine condition. In contrast, syllable spectral purity was highest when they sang to awake females (FE; Figure 5E; Table S2E), indicating that songs emitted in the awake female condition are sharper than in all the other contexts. The fact that pattern of spectral purity is not an entirely inverse pattern of bandwidth, is consistent with our calculations of spectral purity and bandwidth measuring different aspects of syllable structure.

### Males sing their longest sequences in the presence of an awake female

**Figure 7:**
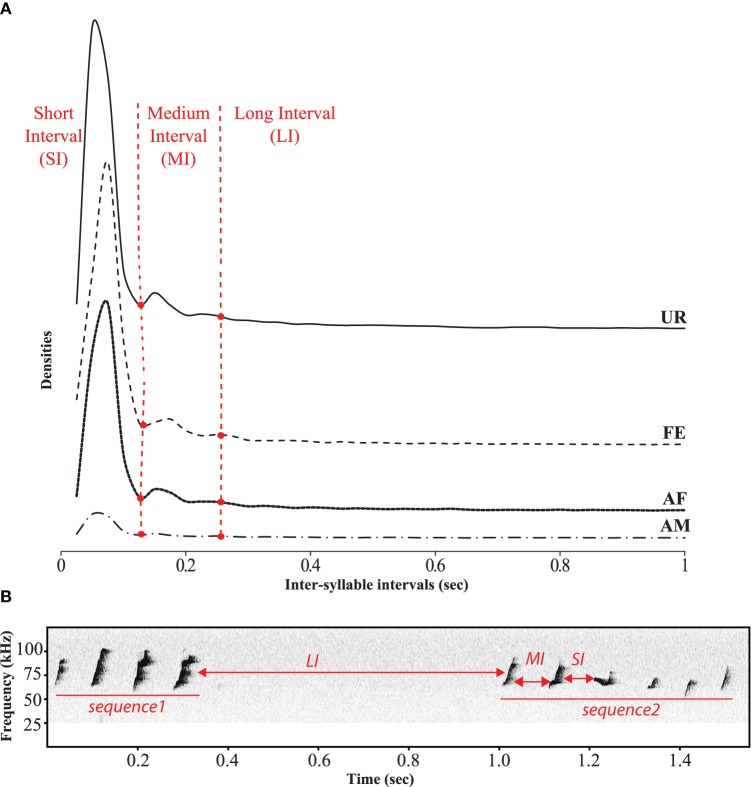
**Temporal organization of sequences in different context**. **(A)** Distribution of the inter-syllables intervals for the four conditions defining three types of silent intervals between sequences of syllables. **(B)** Representation of these three types of intervals on a sonogram separating two songs by a long interval.

In order to identify sequences of songs, we followed a previous approach of analyzing the inter-syllable intervals (ISI) (Ey et al., 2013; Von Merten et al., 2014). However, instead of making an arbitrary cutoff (300 and 500 ms) to determine the silent gap between sequences, we quantified them and were able to identify three comparable categories of ISIs or gaps (Figure 7). The first and most dominant category contained very short intervals (SI) of 0–0.125 s between syllables of a song. The second category consisted of medium length intervals (MI) of 0.125–0.250 s, also between syllables of a song. The lower bound of this category was taken as two times the variance of the second peak (+2 ∂, thus having 95% confidence intervals that this interval differs from the short one). We took the cut of the MI interval at that 3rd peak (2nd low peak) to capture as much of this interval, which was also more than +2 ∂ (two standard deviations from the center of the peak), and matched the trough of an even smaller peak, defining the third category. It consisted of longer inter-syllable intervals (LI) of more than 0.250 s, which separated different songs within a bout of singing. Considering these three categories, we defined a sequence of syllables or song as a succession of syllables separated by SI or MI, whereas song bouts were separated by LI. The distribution profiles of the inter-syllable intervals (ISI) had a similar shape across social context (Figure 7).

Based on our above definition of a sequence, we analyzed sequence lengths (i.e., the number of consecutive syllables separated by a short or a medium interval) in a song bout. We found that consistent with the amount of singing, males emitted longer sequences when exposed to an awake female, followed by an anesthetized female and urine conditions (Figure 5F; Table S2F).

### Syntax differed between context

**Figure 8:**
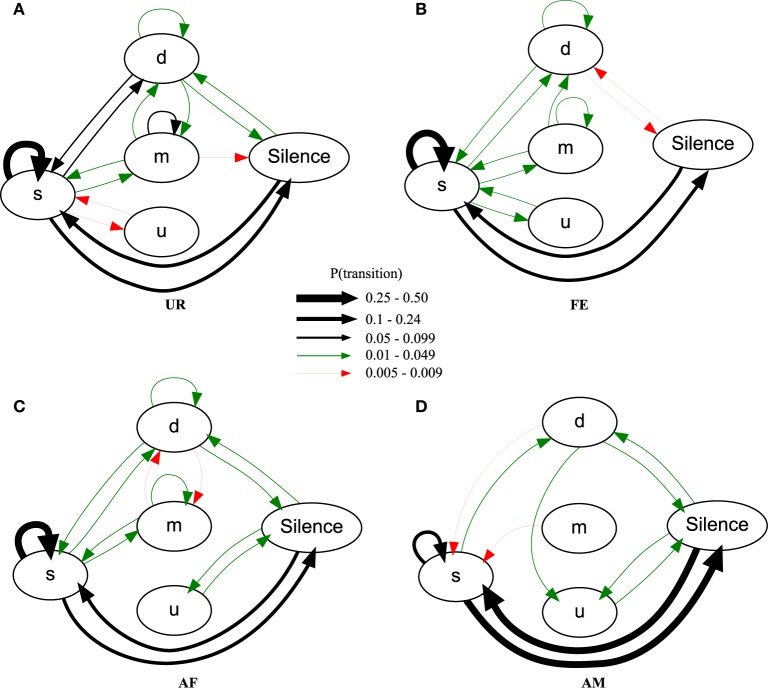
**Graphical representation of sequences of general syllable transition probabilities for each context**. Diagrams are representations of the overall probability of occurrence of a transition type in **(A)** UR, **(B)** FE, **(C)** AF and **(D)** AM conditions. Each arrow between syllable types represent the probabilities of going from one syllable type to another, averaged from *n* = 12 males. Thickness is proportional to the P (occurrence of a transition) value. For clarity, we only represent probabilities higher than 0.005 (higher than 0.5% of chance of occurrence).

Using the general probability of occurrence model (PO), we found across males, consistently different sequence organizations in all four contexts [MANOVA Pillai's Trace: *F*_(72, 69)_ = 1.953, *p* = 0.003; Figure 8; Table S4; taking into account only transition probabilities equal or higher than 0.005]. The syllable type most likely to start a sequence in all the contexts was the simple “s” type (Figure 8, respectively UR: 83.4 ± 2.9%; AF: 83.1 ± 3.4%; FE: 89.7 ± 1.5%; AM: 87 ± 6%) explaining the high overall probability of the transition between Silence and “s.” In the UR, FE, and AF contexts, the “s,” “m,” and “d” syllables were repeated in loops (in succession), but the “u” type was not; in the AM context, only the “s” type was repeated in loops. The most common successive repetition was with the simple “s” syllable type, and the most common inter-syllable transition was “s” with silence in AM (31% of the time), and “s” to “s” for all the others (UR: 32%, AF: 38%, FE: 49% of the time).

There were greater syntax similarities between the UR and FE contexts, and between the AM and AF contexts (Figure 8). For example, the AM condition had 10 of its 11 transitions (91%) in common with the AF condition, while only 8 (73%) in common with UR and FE conditions. Conversely, the FE condition had 14 of its 14 transitions (100%) in common with the UR condition, while only 11 (79%) and 9 (64%) in common with AF and AM conditions. For specific transition differences among contexts, the “u” was linked with “s” in UR and FE context, but only linked with silence in AM and AF. In the UR condition we observed higher probabilities of transitions between “s” and the rest of the repertoire [black arrows, *P*(transition) ≥ 0.05, for “m” loops and other transitions, and “d” and “s” transitions] compared to FE and AF contexts, making the latter sequences more “s-like” [green arrows, *P*(transition) ≤ 0.049]. Songs in the AM context were more limited to only reciprocal transitions, meaning AM is more linear than all others. Although the total number of transition types was close among contexts, the male mice produced more transition types (16) and more often with the more complex syllable type (m) in the urine (UR) context, than in the FE (14) and AF (15) contexts, which in turn was more than in the AM (11) context. This suggest that like the syllables, syntax maybe most complex in the UR condition.

**Figure 9:**
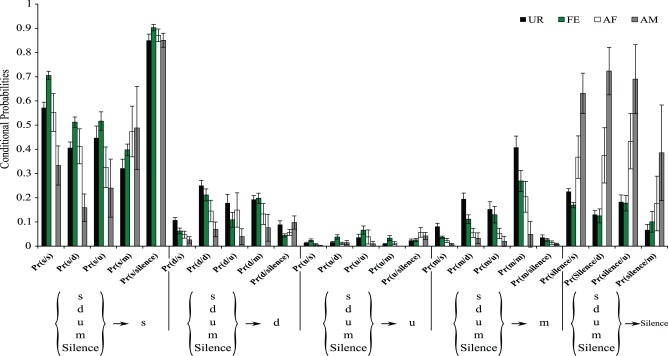
**Conditional probabilities of occurrence for each context**. Bar graphs represent the conditional probabilities of occurrence of a transition type in each condition averaged from *n* = 12 males. Data are presented for B6D2F1/J male (*n* = 12) mice as mean ± SEM.

It is possible that in the general probability model the “m” syllable type in the UR condition is more interconnected with other syllable types than in other conditions not because of more syntax diversity, but because of the greater probability of the “m” syllable produced in the UR condition (Figure 3B). To help distinguish between these two possibilities, we estimated the conditional probabilities (Figure 9), focusing on transitions with fixed starting syllables, and developed an approach that tests for systematic differences in these probabilities (see Appendix A in Supplementary Material). Under a completely random transition mechanism, the conditional probabilities of choosing a syllable will be proportional to the overall preference for that syllable. However, consistent with the general probability findings, we found this to be not true [T_X^2^_(obs.) = 12,811.28, *df* = 720, *p* < 0.0001; Appendix A in Supplementary Material]; that is, the conditional probabilities Pr(x/d), Pr(x/s), Pr(x/u), Pr(x/m), and P(x/silence) for a given syllable “x” are not proportional in all contexts (Figure 9). Further we found that tests for differences in the distributions of transition types for different starting syllables across contexts were also highly significant (not random), including for the m syllable [T_X^2^_(obs.) = 5167.109, *df* = 684, *p* < 0.0001; Figure 9 and Appendix A in Supplementary Material]; pair-wise comparisons using Chi-squared tests for each pair of contexts showed strong statistical differences (Figure 9 and Appendix A in Supplementary Material: *p* < 0.0001). This supports our first hypothesis, being that in different contexts the mice's choice of the transition types for given starting syllables differs, and that the there is more syntax diversity in the urine condition.

**Figure 10:**
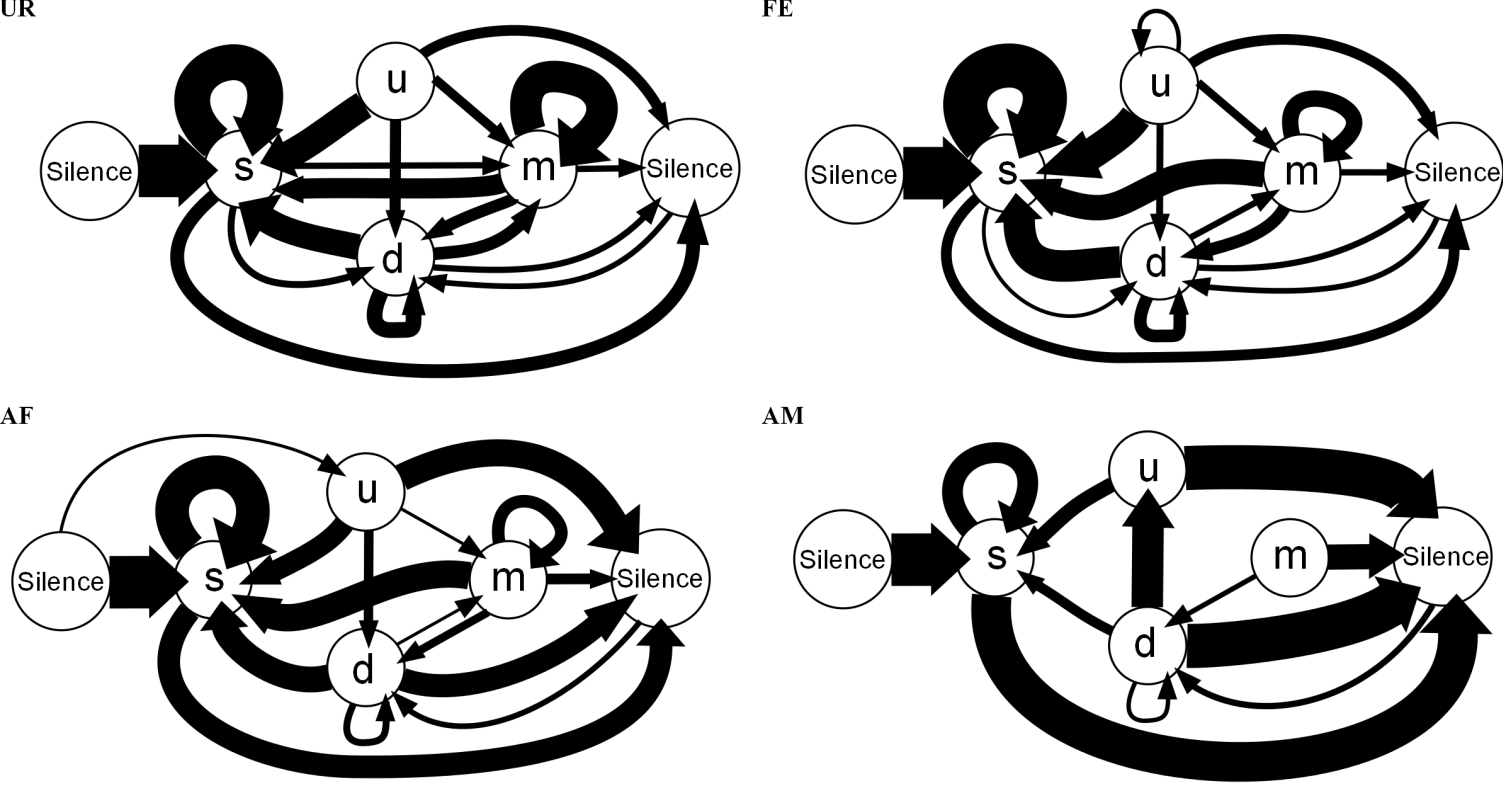
**Graphical representation of sequences of conditional syllable transition probabilities for each context**. Diagrams represent the conditional probability of occurrence (for the probabilities above the threshold of *p* = 0.05) of a transition type in each context averaged from *n* = 12 males. Thickness is proportional to the P (occurrence of a transition given the starting syllable) value. For clarity, rare transitions below a probability of 0.05 are not shown, except the highest probability transition to the “u” syllable type, as all such transitions were below the cut off values. All the values can be seen in Figure 9.

These differences in syntax diversity can be seen in graphical representation of the conditional probabilities in different context (Figure 10). Overall, the conditional song syntax also appeared to have the most complexity (diversity) in the urine context, followed by the awake female and then anesthetized female contexts, and the least complexity in the anesthetized male context. Fresh female urine triggered long sequences starting with simple syllables (type “s,” without frequency jumps) and then mainly complex syllables with frequency jumps (types d, u, or m), which we call complex sequences. Awake and behaving females triggered slightly longer sequences composed of more simple syllables (type s), with a narrow frequency range and high spectral purity, which we call simple sequences. Anesthetized female triggered sequences relatively closer to the awake female context, but shorter. And anesthetized male condition triggered very simple and short sequences composed of more simple syllables.

### The proportion of complex vs. simple song syllables varied with context

**Figure 11:**
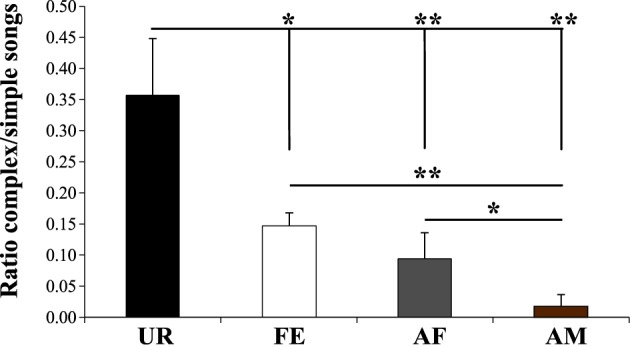
**Ratio of complex songs over simple songs**. Shown is number of sequences with 2 or more complex “m” syllables divided by the number of sequences with 1 or no “m” syllables in each context. Sequences with less than 2 syllables total were not included. Data are presented are mean ± sem. ^*^p < 0.041, ^**^p < 0.005 for *post-hoc* Student's paired *t*-test (*n* = 12 males).

We noted that although the repertoire composition in all contexts was dominated by the simple “s” syllable type (Figure 3B), examination of sonograms of long sequences suggested to us that the proportion of sequences with complex syllables also varied with context. To quantify this difference, we measured the ratio of complex sequences (composed by at least 2 occurrences of the “m” syllable type) vs. simple sequences (composed of one or no “m” types, and thus mostly by “s” type). As suggested by the sonograms and syntax diagrams, we observed that male mice exposed to female urine (UR) produced 2.3X–15X higher ratios of sequences with complex “m” syllables relative to other contexts (Figure 11; Table S5). Indeed, in each of the FE, AF, and AM contexts, the males produced successively fewer sequences with 2 or more “m” syllables. This finding indicates that not only are there more complex syllables produced in the urine context, but also that such syllables are distributed over more sequences, whereas in the other context, fewer complex syllables are restricted to proportionally fewer sequences.

### Females detect differences in song of different context and show preferences

**Figure 12:**
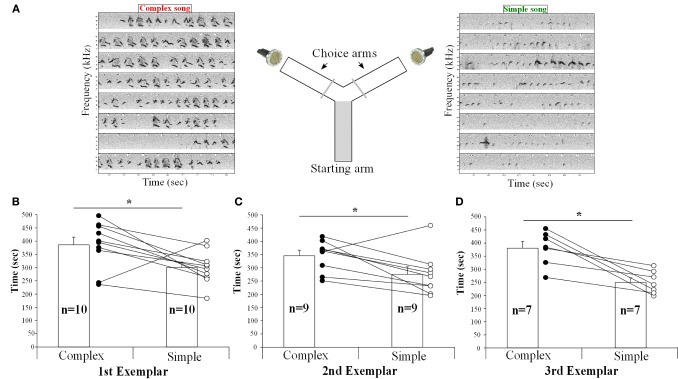
**Female choices between playbacks of complex and simple songs**. **(A)** Schematic representation of the behavioral paradigm used. **(B)** Time (in seconds) spent by the females in each arm playing either the UR or FE song from the first, **(C)** second, **(D)** and third exemplar males. *Paired t-test: first t9* = *2.65, p* = *0.02; second: t8* = *2.67, p* = *0.028; third: t6* = *4.38, p* = *0.004*. Data are presented for B6D2F1/J female mice as mean ± SEM. ^*^p < 0.05 for Student's *t*-test.

To determine if the females could detect and thus show a preference for song from different contexts, we placed them in a Y-maze choice test and simultaneously played back songs of the same males from the urine and the awake female contexts in each arm of the Y maze, controlling for song duration and loudness (Figure 12A). The female urine stimulated songs (from UR) from three males contained a majority of “d,” “u,” and “m” syllables, whereas their awake and behaving or anesthetized female-stimulated songs (from FE and AF) contained mostly simple “s” syllables (Figures 12, S2, S3). We found that one female in the second male exemplar test, and three females in the third exemplar test had strong side biases (chose one side >75% of the time, regardless of song, even after re-testing). It seems that the side bias of females increases with the number of test sessions. Analyzing all sessions without a side bias (*n* = 10, *n* = 9, and *n* = 7 females, for male examples 1, 2, and 3, respectively), we found that nearly all females spent more time (on average ~30% more) in the arm which had the complex stimulated urine song from all three males than in the arm with the simple song that was played simultaneously (Figures 12B–D). However, two different females showed the opposite preference for one exemplar song pair each (Figures 12B–C). We believe that the females show a preference rather than an avoidance of the other song, because although they had the choice to return to the arm of the Y maze without song playbacks, they instead went back and forth in the arm with the two songs and chose one of them more often. These preferences could easily be seen in individual animals, where from one session to the next as the side of complex song was switched, so did the female go and switch the amount of time spent near the speaker for that song.

## Discussion

In this study we found that B6D2F1/J male mice modified their repertoire composition and syntax in different contexts, and that this difference matters for the listening females. The greater amount of song produced with female-related stimuli is consistent with the hypothesis that some USVs are used as courtship songs to attract the females (Holy and Guo, 2005; Hammerschmidt et al., 2009; Sugimoto et al., 2011; Yang et al., 2013). We suggest that mice exposed to different social stimuli change their songs based on their relative value for social/sexual rank, and likelihood of attracting a female. In this regard, we were surprised to find that the male mice produced more complex songs in response to female urine only compared to live females, and that the females seemed to prefer these songs produced in their absence. One possible explanation that the more complex song is a “calling song” intended to attract females when males can't see but can smell the female, and he believes she is nearby. Then, when the female is present, the male does not need to “call” her anymore and he switches to a more simple, stereotyped, and possibly less energetic, song while trying to keep up with and follow her. The fact that the males are singing louder when exposed to the fresh female urine is suggestive evidence for a “calling song.” Below we present our broader interpretations from the male and female's perspectives.

### The male perspective

Several studies have shown modification of male mouse songs based on experience (Wang et al., 2008; Chabout et al., 2012; Hanson and Hurley, 2012). A more recent study also found differences in repertoire composition between French and German wild mice exposed to different social conditions (Von Merten et al., 2014). The French and German wild mice used complex syntax but the authors did not determine if there were syntax changes in different social contexts. The sequences of vocalizations elicited also appear much shorter, closer to what we find with anesthetized males. The modification of the song syntax we found highlights an important degree of short-term vocal variability or song type switching in mice. Indeed, in the presence of female urine, after one to three utterances of the simple syllable, males shift to more complex syllables and sequences. In the presence of a live female, the male emits longer simple syllables and sequences, before switching to more complex ones, and has a more tonal consistent pitch.

These differences are reminiscent of some findings in songbirds. In a number of songbird species, males produce learned songs mainly for territorial defense or courtship (Morris, 1954). Similarly, two ways of singing in different social contexts have been described in the most commonly studied songbird, the zebra finch (Morris, 1954; Dunn and Zann, 1996; Jarvis et al., 1998): (1) undirected song, which seems to not be addressed to any one bird since the male is usually alone when singing; and (2) courtship directed song, which is usually produced directly facing a female. The undirected song is more variable in pitch and sequence and is thought to be used for practice; the directed song is more consistent in pitch, is sung faster, has more introductory simpler notes, and is presumably used to catch the female's attention and bring them close for mating (Jarvis et al., 1998; Kao and Brainard, 2006; Woolley and Doupe, 2008). We believe that the urine only and live female context-dependent differences in mouse songs are reminiscent of these differences in zebra finches, and other songbirds. This would suggest that such context differences are not limited to vocal learning species, as we note here for other contextual use of vocalizations found in vocal learners [bats (Bohn et al., 2013), dolphins (Janik, 2000), and humans (Kuhl et al., 1997; Doupe and Kuhl, 1999)] and vocal non-learners [frogs (Chakraborty et al., 2010; Kime et al., 2010), chickens (Marler et al., 1986; Karakashian et al., 1988)]. Nevertheless, our findings reveal the possibility that the multisyllabic nature of the mouse vocalizations and their switching to different song types can serve, at least, as a model behavior of social context differences that have been described in songbirds.

We do not know the brain or genetic mechanisms that control the song production differences in contexts in male mice. In the male zebra finch, the undirected and directed songs are associated with different levels and types of neural and gene activity of an anterior forebrain vocal pathway (Jarvis et al., 1998; Hessler and Doupe, 1999). This pathway includes cortical-like and basal ganglia song nuclei, which are more highly activated and show more variable activity when the bird sings undirected song relative to directed song. Analogous, but more rudimentary, vocal regions have been proposed in mice that include primary and secondary motor cortex and a striatal region (Jarvis, 2004b; Arriaga and Jarvis, 2013). Also, several studies show evidence that the sequences of male vocalizations are under strong genetic control (Choi et al., 2011; Kikusui et al., 2011). Future investigations can determine whether these brain regions in mice are differentially active, modulate syllable variability in different social contexts, and whether the genetic loci that affect sequences are expressed in these or other brain regions involved in communication.

### The female perspective

Several previous playback studies showed female mice approach male USVs (Shepard and Liu, 2011; Asaba et al., 2014b), and prefer natural songs emitted by a male over computer-generated songs (Hammerschmidt et al., 2009). A more recent study used a slightly different playback setup to show that females can discriminate among male song characteristics and prefer songs of mice that are different from their parents (Asaba et al., 2014b). However, no distinction has been made between the different kinds of songs male mice produce. Our playback experimental results suggest that the more complex songs are more attractive to the females. We suggest that in the wild, under natural context, the male could be producing these more complex vocalizations very soon after a female urinates in his vicinity within ear shot, as a mechanism to attract her closer. Once she is in visible site, he switches to the more simple song. Since the complex songs are composed mostly of frequency jumps and more highly frequency modulated syllables (such as “d,” “u,” or “m”), we assume that some of these syllables or at least some of their spectral features are appealing to the female, as is the case in some birds where some more highly modulated song elements can make a difference in terms of how attractive it is (Rehsteiner et al., 1998).

In zebra finches, females prefer the more stereotyped directed song in terms of syllable pitch (Woolley and Doupe, 2008), but in certain other songbird species, such as the canary, females show a high level of interest by increasing their sexual displays when exposed to playbacks of the less stereotyped more modulated sexy “A” syllables from males, which contain two simultaneous different notes (Vallet and Kreutzer, 1995; Zann, 1996; Vallet et al., 1998; Marshall et al., 2005). Female tùngara frogs are also more attracted to complex songs (although relatively simple compared to mice) containing a specific additional note, called “chucks” (Chakraborty et al., 2010; Kime et al., 2010). These results are consistent with our findings in mice, where the females prefer the song with more complex syllables and sequences.

## Conclusions

Male mice appear to be producing specific syllables according to a non-random mechanism. Males have multiple song variants, and they use them differently depending on the context. The song differences have meaning for the female and can attract her to the male. Our hypothesis of a calling song can be tested with playbacks using different syllable types and sequences, measuring the female hormonal responses and also by studying the males singing behavior in a more open field environment, where the female has the ability to go in and out of view to the male. The differences between the song types and contexts are reminiscent of undirected and directed singing in birds, but there are still many differences and more need to be explored. Examining the details and differences of these songs can reveal novel insights into the role of USVs in mice and study of their use may provide unique purviews into diseases affecting vocal communication.

### Conflict of interest statement

The authors declare that the research was conducted in the absence of any commercial or financial relationships that could be construed as a potential conflict of interest.

## Supplementary material

Figure S1 **Examples of unclassified sounds. (A)** Mechanical noise made by mouse in the cage. **(B)** Machine noise in the room, recorded during opening the cage to put in the cotton tip in the cage with female urine. **(C)** Syllable overlap with mechanical noise made by the mouse. **(D)** A true unclassified syllable, which contains more complex harmonic structure.

Figure S2 **Sonogram of simple and complex songs from the second exemplar**.

Figure S3 **Sonogram of simple and complex songs from the third exemplar**.
