# Supplementary figures and images for: Male mice song syntax depends on social contexts and influences female preferences
Source: Front Behav Neurosci. 2015 Apr 1;9:76. doi: 10.3389/fnbeh.2015.00076 (PMC4383150; doi:10.3389/fnbeh.2015.00076)

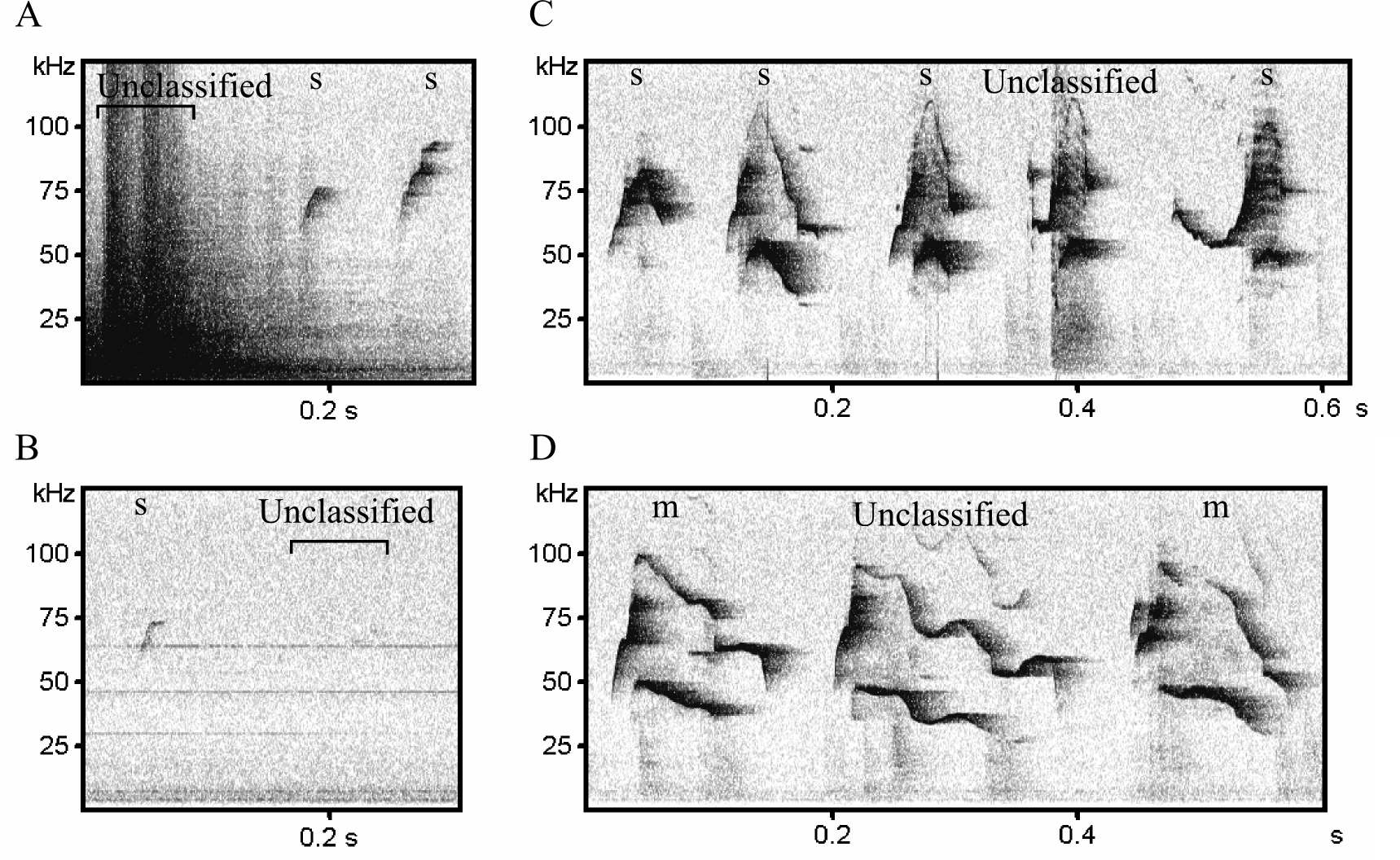

Supplement: Figure S1 — Examples of unclassified sounds. (A) Mechanical noise made by mouse in the cage. (B) Machine noise in the room, recorded during opening the cage to put in the cotton tip in the cage with female urine. (C) Syllable overlap with mechanical noise made by the mouse. (D) A true unclassified syllable, which contains more complex harmonic structure. [file FigureS1.TIFF]

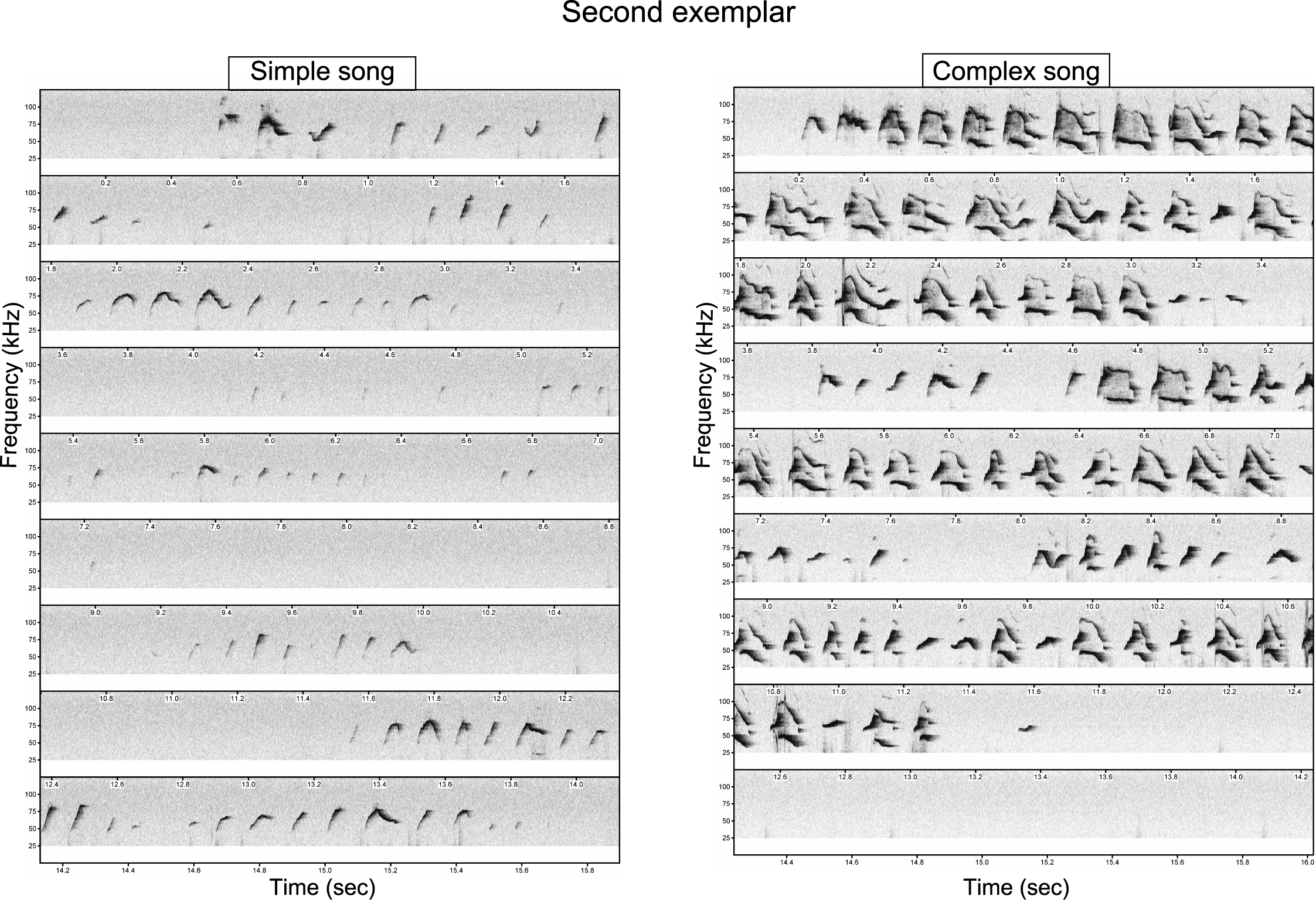

Supplement: Figure S2 — Sonogram of simple and complex songs from the second exemplar. [file FigureS2.TIF]

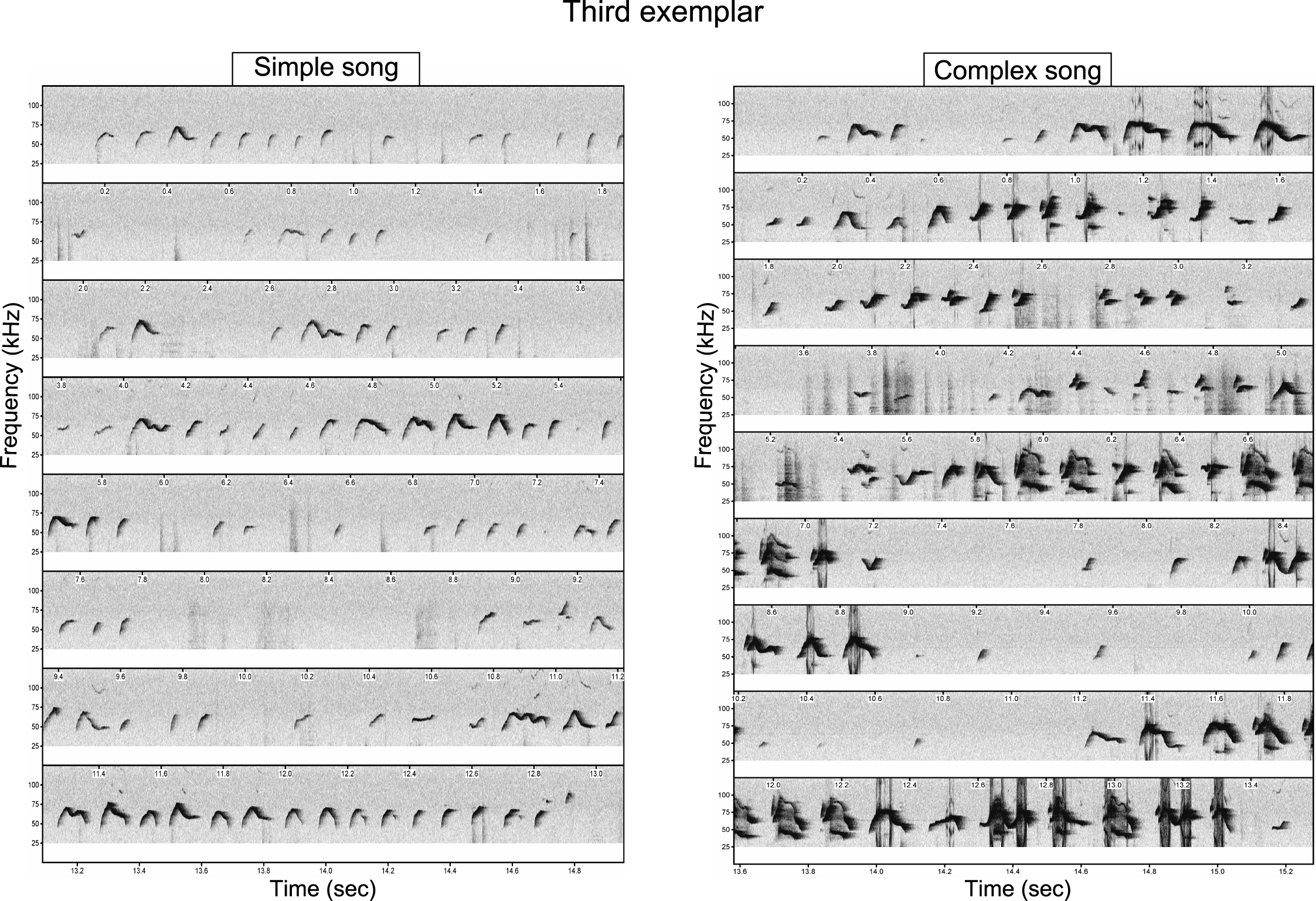

Supplement: Figure S3 — Sonogram of simple and complex songs from the third exemplar. [file FigureS3.TIF]
