# Supplementary material for: Male mice song syntax depends on social contexts and influences female preferences
Source: Front Behav Neurosci. 2015 Apr 1;9:76. doi: 10.3389/fnbeh.2015.00076 (PMC4383150; doi:10.3389/fnbeh.2015.00076)
Supplement: Supplementary file 4 [file TableS1.DOCX]

**Table 1.** Statistical results for **(A)** number of syllables per minute. Condition effect (One way repeated measured ANOVA, df _between_/ df _error_) and for paired comparisons (paired t-test of student). **(B)** Statistical results for repertoire composition, Global effect (MANOVA), then condition effect (One way repeated measured ANOVA, df _between_/ df _error_) and for paired comparisons (paired t-test of student). Significance threshold after Benjamini and Hochberg correction is detailed in each table.

| **(A)** | ***N° of syllable/min*** *(condition effect, Greenhouse-Geisser: F(_2.1_,_23.14_)= 18.82, p<0.0001)* | | | |  |
| --- | --- | --- | --- | --- | --- |
| *Corrected threshold : p=0.03* | FE (N=12) | AF (N=12) | AM (N=12) | URM (N=12) | |
| UR (N=12) | t=0.57, p=0.57 | t=2.2, p=0.045 | t=6.9, p<0.0001 | t=-7.16, p<0.0001 | |
| FE (N=12) | - | t=-1.11, p=0.288 | t=7.1, p<0.0001 | t=-8.92, p<0.0001 | |
| AF (N=12) | - | - | t=3.15, p=0.009 | t=-3.5, p=0.005 | |
| AM (N=12) | - | - | - | t=-2.02, p=0.067 | |

|  |  |  |  | |
| --- | --- | --- | --- | --- |
| **(B)** | ***Repertoire composition*** *(MANOVA Pillai’s Trace: F(_12_,_129_)= 12, p=0.001)*  ***“s”*** *(condition effect, F(_3_,_33_)= 16.21, p<0.0001)* | | |  |
| *Corrected threshold : p=0.03* | FE (N=12) | AF (N=12) | AM (N=12) | |
| UR (N=12) | t=-3.7, p=0.003 | t=-5.29, p=0.0003 | t=-4.9, p=0.0004 | |
| FE (N=12) | - | t=1.13, p=0.28 | t=-3.03, p=0.01 | |
| AF (N=12) | - | - | t=-1.1, p=0.29 | |
|  |  |  |  | |
|  | ***“d”*** *(condition effect, F(_3_,_33_)= 5.77, p=0.002)* | | |  |
| *Corrected threshold : p=0.025* | FE (N=12) | AF (N=12) | AM (N=12) | |
| UR (N=12) | t=4.2, p=0.0015 | t=4.8, p=0.0005 | t=2.83, p=0.01 | |
| FE (N=12) | - | t=-0.56, p=0.58 | t=0.38, p=0.70 | |
| AF (N=12) | - | - | t=-0.16, p=0.86 | |

|  | ***“u”*** *(condition effect, F(_3_,_33_)= 1.23, p=0.31)* | | |  |
| --- | --- | --- | --- | --- |
|  | FE (N=12) | AF (N=12) | AM (N=12) | |
| UR (N=12) | NA | NA | NA | |
| FE (N=12) | - | NA | NA | |
| AF (N=12) | - | - | NA | |
|  | ***“m”*** *(condition effect, F(_3_,_33_)=21.154, p<0.0001)* | | |  |
| *Corrected threshold : p=0.03* | FE (N=12) | AF (N=12) | AM (N=12) | |
| UR (N=12) | t=3.37, p=0.006 | t=6.17, p<0.0001 | t=5.9, p=0.0001 | |
| FE (N=12) | - | t=-1.85, p=0.09 | t=4.43, p=0.001 | |
| AF (N=12) | - | - | t=1.76, p=0.10 | |
