# Supplementary material for: Male mice song syntax depends on social contexts and influences female preferences
Source: Front Behav Neurosci. 2015 Apr 1;9:76. doi: 10.3389/fnbeh.2015.00076 (PMC4383150; doi:10.3389/fnbeh.2015.00076)
Supplement: Supplementary file 5 [file TableS2.DOCX]

**Table 2.** Statistical results for **(A)** Global amplitude **(B)** Syllable duration **(C)** Pitch (mean frequency), **(D)** Bandwidth, **(E)** Spectral purity, **(F)** Sequence length. Condition effect (One way repeated measured ANOVA, df _between_/ df _error_) and for paired comparisons (Student’s paired t-test). Significance threshold after Benjamini and Hochberg correction is detailed in each table.

| **(A)** | ***Global amplitude*** *(condition effect, Greenhouse-Geisser: F(_1.6_,_17.64_)= 7.05, p=0.008)* | | | |
| --- | --- | --- | --- | --- |
| *Corrected threshold : p=0.025* | FE (N=12) | AF (N=12) | AM (N=12) | |
| UR (N=12) | t=3.24, p=0.008 | t=2.77, p=0.018 | t=2.8, p=0.017 | |
| FE (N=12) | - | t=-0.7, p=0.461 | t=-0.27, p=0.78 | |
| AF (N=12) | - | - | t=0.45, p=0.65 | |
| **(B)** | ***Syllable duration*** *(condition effect: F(_3_,_33_)= 26.78, p<0.0001)* | | |  |
| *Corrected threshold : p=0.041* | FE (N=12) | AF (N=12) | AM (N=12) | |
| UR (N=12) | t=1.79, p=0.01 | t=5.76, p<0.0001 | t=9.93, p<0.0001 | |
| FE (N=12) | - | t=3.5, p=0.005 | t=6.22 p<0.0001 | |
| AF (N=12) | - | - | t=2.352, p=0.038 | |

| **(C)** | ***Pitch (frequency mean)*** *(condition effect: F(_3_,_33_)= 27.67, p<0.0001)* | | | |  |
| --- | --- | --- | --- | --- | --- |
| *Corrected threshold : p=0.033* | FE (N=12) | AF (N=12) | AM (N=12) | | |
| UR (N=12) | t=7.9, p<0.0001 | t=2.82, p=0.01 | t=3.73, p=0.003 | | |
| FE (N=12) | - | t=-1.5, p=0.160 | t=2.74, p=0.01 | | |
| AF (N=12) | - | - | t=1.45, p=0.17 | | |
|  |  |  |  | | |
| **(D)** | ***Bandwidth*** *(condition effect: F(_3_,_33_)= 27.676, p<0.0001)* | | | |  |
| *Corrected threshold : p=0.041* | FE (N=12) | AF (N=12) | AM (N=12) | | |
| UR (N=12) | t=6.75, p<0.0001 | t=7.9, p<0.0001 | t=8.9, p<0.0001 | | |
| FE (N=12) | - | t=-1.6, p=0.12 | t=4.13, p=0.001 | | |
| AF (N=12) | - | - | t=2.36, p=0.03 | | |
|  |  | | | | |
| **(E)** | ***Spectral purity*** *(condition effect: F(_3_,_33_)= 9.24, p=0.001)* | | | |  |
| *Corrected threshold : p=0.025* | FE (N=12) | AF (N=12) | AM (N=12) | | |
| UR (N=12) | t=-10.62, p<0.0001 | t=-0.39, p=0.70 | t=1.4, p=0.18 | | |
| FE (N=12) | - | t=-3.66, p=0.003 | t=4.5, p=0.0009 | | |
| AF (N=12) | - | - | t=1.55, p=0.14 | | |
| **(F)** | ***Sequence length*** *(condition effect: F(_3_,_33_)= 14.01, p<0.0001)* | | |  |  |
| *Corrected threshold : p=0.025* | FE (N=12) | AF (N=12) | AM (N=12) | | |
| UR (N=12) | t=-2.26, p=0.04 | t=1.56, p=0.14 | t=6.8, p<0.0001 | | |
| FE (N=12) | - | t=-2.8, p=0.017 | t=6.3, p<0.0001 | | |
| AF (N=12) | - | - | t=2.28, p=0.043 | | |
