# Supplementary material for: Male mice song syntax depends on social contexts and influences female preferences
Source: Front Behav Neurosci. 2015 Apr 1;9:76. doi: 10.3389/fnbeh.2015.00076 (PMC4383150; doi:10.3389/fnbeh.2015.00076)
Supplement: Supplementary file 6 [file TableS3.DOCX]

**Table 3.** Statistical results for **(A)** amplitude of the ‘s’ syllables’, ‘d’ syllable, ‘u’ syllable and ‘m’ syllable across contexts. **(B)** Duration of the ‘s’ syllables’, ‘d’ syllable, ‘u’ syllable and ‘m’ syllable across contexts. **(C)** Pitch (frequency mean) of the ‘s’ syllables’, ‘d’ syllable, ‘u’ syllable and ‘m’ syllable across contexts. Global effect (MANOVA), then Condition effect (One way repeated measured ANOVA, df _between_/ df _error_) and paired comparisons (paired t-test of student). Significance threshold after Benjamini and Hochberg correction is detailed in each table.

| **(A)** | ***Amplitude*** *(MANOVA Pillai’s Trace: F(_12,105_)=1.39 , p=0.179)*  ***Amplitude “s”*** *(condition effect, Greenhouse-Geisser: F(_1.71_,_18.87_)= 4.46, p=0.03)* | | |  |
| --- | --- | --- | --- | --- |
| *Corrected threshold: p=0.0083* | FE (N=12) | AF (N=12) | AM (N=12) | |
| UR (N=12) | t=2.84, p=0.01 | t=2.16, p=0.053 | t=2.09, p=0.061 | |
| FE (N=12) | - | t=-1.07, p=0.30 | t=-0.66, p=0.52 | |
| AF (N=12) | - | - | t=0.03, p=0.97 | |
|  |  |  |  | |
|  | ***Amplitude “d”*** *(condition effect, Greenhouse-Geisser: F(_1.33_,_14.64_)= 4.5, p=0.042)* | | |  |
| *Corrected threshold : p=0.0083* | FE (N=12) | AF (N=12) | AM (N=12) | |
| UR (N=12) | t=2.25, p=0.046 | t=2.17, p=0.052 | t=2.3, p=0.042 | |
| FE (N=12) | - | t=0.99, p=0.34 | t=1.01, p=0.33 | |
| AF (N=12) | - | - | t=0.54, p=0.59 | |

|  | ***Amplitude “u”*** *(condition effect, Greenhouse-Geisser: F(_1.2_,_13.25_)= 3.2, p=0.091)* | | |  |
| --- | --- | --- | --- | --- |
|  | FE (N=12) | AF (N=12) | AM (N=12) | |
| UR (N=12) | NA | NA | NA | |
| FE (N=12) | - | NA | NA | |
| AF (N=12) | - | - | NA | |
|  | ***Amplitude “m”*** *(condition effect, Greenhouse-Geisser: F(_1.67_,_18.39_)= 2.51, p=0.115)* | | |  |
|  | FE (N=12) | AF (N=12) | AM (N=12) | |
| UR (N=12) | NA | NA | NA | |
| FE (N=12) | - | NA | NA | |
| AF (N=12) | - | - | NA | |

| **(B)** | ***Duration*** *(MANOVA Pillai’s Trace: F(_12,105_)=4.98, p<0.0001)*  ***Duration “s”*** *(condition effect, Greenhouse-Geisser: F(_1.906,20.970_)= 16.916, p<0.0001)* | | |  |
| --- | --- | --- | --- | --- |
| *Corrected threshold: p=0.041* | FE (N=12) | AF (N=12) | AM (N=12) | |
| UR (N=12) | t=2.455, p=0.032 | t=3.477, p=0.005 | t=8.39, p<0.0001 | |
| FE (N=12) | - | t=2.395, p=0.036 | t=5.166, p<0.0001 | |
| AF (N=12) | - | - | t=2.11, p=0.058 | |
|  |  |  |  | |
|  | ***Duration “d”*** *(condition effect : F(_3_,_24_)= 16.621, p<0.0001)* | | |  |
| *Corrected threshold : p=0.033* | FE (N=12) | AF (N=12) | AM (N=12) | |
| UR (N=12) | t=-2.08, p=0.061 | t=1.909, p=0.085 | t=8097, p<0.0001 | |
| FE (N=12) | - | t=2.675, p=0.023 | t=8.654, p<0.0001 | |
| AF (N=12) | - | - | t=2.79, p=0.024 | |

|  | ***Duration “u”*** *(condition effect: F(_3_,_24_)= 20.87, p<0.0001)* | | |  |
| --- | --- | --- | --- | --- |
| *Corrected threshold : p=0.041* | FE (N=12) | AF (N=12) | AM (N=12) | |
| UR (N=12) | t=-3.288, p=0.007 | t=3.627, p=0.004 | t=6.306, p<0.0001 | |
| FE (N=12) | - | t=5.708, p<0.0001 | t=5.908, p<0.0001 | |
| AF (N=12) | - | - | t=0.635, p=0.541 | |
|  | ***Duration “m”*** *(condition effect: F(_3_,_24_)= 4.036, p=0.045)* | | |  |
| *Corrected threshold : p=0.033* | FE (N=12) | AF (N=12) | AM (N=12) | |
| UR (N=12) | t=-4.028, p=0.002 | t=1.92, p=0.091 | t=4.07, p=0.007 | |
| FE (N=12) | - | t=3.94, p=0.004 | t=4057, p=0.004 | |
| AF (N=12) | - | - | t=0.173, p=0.873 | |

| **(C)** | ***Pitch*** *(MANOVA Pillai’s Trace: F(_12_,_105_)= 12, p=0.009)*  ***Pitch “s”*** *(condition effect, Greenhouse-Geisser: F(_1.85_,_20.41_)= 7.232, p=0.005)* | | |  |
| --- | --- | --- | --- | --- |
| *Corrected threshold: p=0.041* | FE (N=12) | AF (N=12) | AM (N=12) | |
| UR (N=12) | t=6.63, p<0.0001 | t=3.11, p=0.01 | t=3.67, p=0.004 | |
| FE (N=12) | - | t=-1.785, p=0.10 | t=2.56, p=0.026 | |
| AF (N=12) | - | - | t=1.25, p=0.235 | |
|  |  |  |  | |
|  | ***Pitch “d”*** *(condition effect, Greenhouse-Geisser: F(_1.546_,_17.01_)= 5.6, p=0.019)* | | |  |
| *Corrected threshold : p=0.025* | FE (N=12) | AF (N=12) | AM (N=12) | |
| UR (N=12) | t=4.65, p=0.001 | t=1.76, p=0.10 | t=3.57, p=0.004 | |
| FE (N=12) | - | t=1.094, p=0.297 | t=3.11, p=0.010 | |
| AF (N=12) | - | - | t=1.522, p=0.156 | |

|  | ***Pitch “u”*** *(condition effect, Greenhouse-Geisser: F(_1.46_,_16.136_)= 5.28, p=0.025)* | | |  |
| --- | --- | --- | --- | --- |
| *Corrected threshold : p=0.0083* | FE (N=12) | AF (N=12) | AM (N=12) | |
| UR (N=12) | t=2.23, p=0.047 | t=0.996, p=0.341 | t=3.194, p=0.009 | |
| FE (N=12) | - | t=-0.547, p=0.595 | t=2.12, p=0.057 | |
| AF (N=12) | - | - | t=2.131, p=0.056 | |
|  | ***Pitch “m”*** *(condition effect, Greenhouse-Geisser: F(_1.438_,_15.813_)= 3.954, p=0.052)* | | |  |
| *Corrected threshold : p=0.016* | FE (N=12) | AF (N=12) | AM (N=12) | |
| UR (N=12) | t=2.5, p=0.029 | t=1.856, p=0.090 | t=3.435, p=0.006 | |
| FE (N=12) | - | t=1.423, p=0.182 | t=3.026, p=0.012 | |
| AF (N=12) | - | - | t=0.812, p=0.434 | |
