# Supplementary material for: Male mice song syntax depends on social contexts and influences female preferences
Source: Front Behav Neurosci. 2015 Apr 1;9:76. doi: 10.3389/fnbeh.2015.00076 (PMC4383150; doi:10.3389/fnbeh.2015.00076)
Supplement: Supplementary file 7 [file TableS4.DOCX]

|  | ***Overall probabilities for all transition types*** | | | | | | | | | | | | | | | | | | | | | | | |
| --- | --- | --- | --- | --- | --- | --- | --- | --- | --- | --- | --- | --- | --- | --- | --- | --- | --- | --- | --- | --- | --- | --- | --- | --- |
|  | ***ss*** | ***sd*** | ***su*** | ***sm*** | ***sSil.*** | ***ds*** | ***dd*** | ***du*** | ***dm*** | ***dSil.*** | ***us*** | ***ud*** | ***uu*** | ***um*** | ***uSil.*** | ***ms*** | ***md*** | ***mu*** | ***mm*** | ***mSil.*** | ***Sil.s*** | ***Sil.d*** | ***Sil.u*** | ***Sil.m*** |
| ***UR*** | 0.32  ±  0.02 | 0.05  ±  0.002 | *0.007*  *±*  *0.002* | 0.04  ±  0.005 | 0.12  ±  0.009 | 0.05  ±  0.003 | 0.036  ±  0.005 | 0.002  ±  0.0005 | 0.027  ±  0.005 | 0.017  ±  0.002 | 0.007  ±  0.002 | 0.002  ±  0.0005 | .0007  ±  .0003 | 0.0016  ±  0.0003 | 0.002  ±  0.0006 | 0.04  ±  0.004 | 0.03  ±  0.005 | 0.001  ±  0.0002 | 0.06  ±  0.015 | 0.006  ±  0.001 | 0.13  ±  0.009 | 0.01  ±  0.002 | 0.003  ±  0.001 | 0.004  ±  0.001 |
| ***FE*** | 0.49  ±  0.02 | 0.04  ±  0.007 | 0.015  ±  0.003 | 0.025  ±  0.0027 | 0.12  ±  0.008 | 0.04  ±  0.006 | 0.02  ±  0.004 | 0.002  ±  0.0007 | 0.01  ±  0.0017 | 0.008  ±  0.0015 | 0.014  ±  0.0035 | 0.0029  ±  0.0007 | 0.0027  ±  0.001 | 0.003  ±  0.001 | 0.0033  +  0.0005 | 0.023  ±  0.0028 | 0.012  ±  0.0021 | 0.0021  ±  0.0007 | 0.02  ±  0.0047 | 0.0037  ±  0.0005 | 0.12  ±  0.0083 | 0.0059  ±  0.0008 | 0.003  ±  0.0008 | 0.0036  ±  0.0008 |
| ***AF*** | 0.38  ±  0.066 | 0.03  ±  0.0086 | 0.004  ±  0.002 | 0.014  ±  0.0043 | 0.20  ±  0.034 | 0.029  ±  0.0086 | 0.015  ±  0.006 | 0.0008  ±  0.0003 | 0.006  ±  0.003 | 0.017  ±  0.0055 | 0.0045  ±  0.0019 | 0.0028  ±  0.002 | 0.0011  ±  0.0008 | 0.001  ±  0.0007 | 0.017  ±  0.009 | 0.011  ±  0.004 | 0.007  ±  0.0033 | 0.0006  ±  0.0003 | 0.01  ±  0.005 | 0.0042  ±  0.0025 | 0.20  ±  0.035 | 0.013  ±  0.004 | 0.020  ±  0.009 | 0.0018  ±  0.0007 |
| ***AM*** | 0.21  ±  0.057 | 0.013  ±  0.006 | 0.0015  ±  0.0007 | 0.004  ±  0.003 | 0.316  ±  0.028 | 0.0084  ±  0.0041 | 0.0035  ±  0.002 | 0.0004  ±  0.0002 | 0.0022  ±  0.0017 | 0.039  ±  0.012 | 0.0025  ±  0.0013 | 0.0003  ±  0.0013 | 0.0001  ±  0.0001 | 0.0001  ±  0.0001 | 0.015  ±  0.0064 | 0.0055  ±  0.0034 | 0.0017  ±  0.0016 | 0  ±  0 | 0.0026  ±  0.0027 | 0.0026  ±  0.0014 | 0.31  ±  0.028 | 0.04  ±  0.01 | 0.017  ±  0.006 | 0.0033  ±  0.0016 |

**Table 4.** Overall probabilities for all transition types across contexts.

Abbreviation Sil. = silence, or ISI longer than 250ms.
