# Supplementary material for: Male mice song syntax depends on social contexts and influences female preferences
Source: Front Behav Neurosci. 2015 Apr 1;9:76. doi: 10.3389/fnbeh.2015.00076 (PMC4383150; doi:10.3389/fnbeh.2015.00076)
Supplement: Supplementary file 8 [file TableS5.DOCX]

**Table 5.** Statistical results for ratio of complex songs over simple songs. Condition effect (One way repeated measured ANOVA, df _between_/ df _error_) and for paired comparisons (paired t-test of student). Significance threshold after Benjamini and Hochberg correction was set at p<0.041.

|  | ***Ratio C/S*** *(condition effect, Greenhouse-Geisser: F(_1.62_,_16.27_)= 12.09, p=0.001)* | | |  |
| --- | --- | --- | --- | --- |
| *Corrected threshold : p=0.041* | FE (N=12) | AF (N=12) | AM (N=12) | |
| UR (N=12) | t=2.44, p=0.03 | t=3.75, p=0.003 | t=4.7, p=0.0006 | |
| FE (N=12) | - | t=-1.4, p=0.18 | t=4.9, p=0.0004 | |
| AF (N=12) | - | - | t=2.48, p=0.033 | |
