# Supplementary material for: Male mice song syntax depends on social contexts and influences female preferences
Source: Front Behav Neurosci. 2015 Apr 1;9:76. doi: 10.3389/fnbeh.2015.00076 (PMC4383150; doi:10.3389/fnbeh.2015.00076)
Supplement: Supplementary file 9 [file AppendixA.PDF]

Appendix A: Tests for Transition Probabilities

In this Appendix, we describe tests for random syllable transition dynamics against a simple first order Markov alternative and summarize the results. The main goal of the tests is to show that the syllable generation mechanism in male mice under different stimuli is not completely random. We assumed a first order Markov model for the syllable transitions as a simple and parsimonious local alternative hypothesis that is powerful to a broad class of sequential dependence structures. Even if the true data generating model is a higher order Markov model, a test of the completely random null hypothesis against a simple first order alternative often provides strong evidence against the null. In simulation experiments, we generated data using second and third order Markov models and tested the null hypothesis of complete randomness against a first order Markov alternative. In all the cases tested, the distribution of the p-values were very highly concentrated near zero. For instance, when the true data generating model was a second order Markov model, the null hypothesis of complete randomness was rejected at the 5% and 0.5% levels of significance approximately 97.4% and 97.1% of the times for a single chain comprising 4 states and 500 data points.

Next, consider the problem of comparing different contexts. In this case, when the true data generating mechanisms are actually higher order Markov, under the null hypothesis of equality of the mechanisms, their first order approximations are also expected to be close, and under the alternative hypothesis of different mechanisms, their first order approximations are also expected to be different. Therefore, in this case also, tests based on first order Markov models will be powerful for a broader class of higher order alternatives. In simulations, when we generated two chains each comprising 4 states and 500 data points using two different second order Markov models and tested the null hypothesis of equality of the transition mechanisms under a first order assumption, the null hypothesis was rejected at the 5% and 0.5% levels approximately 99.2% and 98.6% of the times. And under the null, when we used the same second order mechanism to generate the two chains, the test based on the first order approximation resulted in p-values that were greater than 0.05 87.3% of the time.

For $i = 1, \ldots, n; j = 1, \ldots, K; k \in K(j)$; $c \in C$ and $t = 1, \ldots, T_{i,c}$, let $Y_{i,c,t} = k$ if the ith subject at the tth time point under the sth context is at state k. Let $\mathbf{Y}_{i,c} = (Y_{i,c,1}, \ldots, Y_{i,c,T_{i,c}})^{\mathrm{T}}$. Assuming a first order Markov framework for the transition dynamics, the likelihood function is given by

$$L(\{\mathbf{Y}_{i,c}\}_{i=1,c\in C}^{n}, \{\pi_{k|(i,c)}\}_{c\in C,k\in K}, \{\pi_{k|j(i,c)}\}_{c\in C,j\in K,k\in K(j)})$$
$$= \prod_{c\in C}\prod_{i=1}^{n}\left\{\prod_{k\in K}\pi_{k|(i,c)}^{n_{k(i,c)}^{1}}\prod_{j\in K}\prod_{k\in K(j)}\pi_{k|j(i,c)}^{n_{k|j(i,c)}}\right\},$$

where $\pi_{k|j(i,c)} = Pr(Y_{i,c,t} = k \mid Y_{i,c,t-1} = j)$ for all $i = 1, \ldots, n$ and all $t = 2, \ldots, T_{i,c}$; $n_{k|j(i,c)} = \sum_{t=2}^{T_{i,c}} 1(Y_{i,c,t-1} = j, Y_{i,c,t} = k)$, the total number of transitions from syllable j to syllable k by the ith mouse under context c. Also, $\pi_{k|(i,c)} = Pr(Y_{i,c,1} = k)$, the initial distribution probabilities under context c; and $n^1_{k(i,c)} = 1(Y_{i,c,1} = k)$.

For the mice data set, we have $n = 12$, $K = \{d, m, s, u, x\}$, where for notational convenience we represent the state 'silence' by 'x'; $K(j) = K$ for $j = \{d, m, s, u\}$ but $K(x) = \{d, m, s, u\}$; and $S = \{UR, FE, AF, AM\}$. $K(x)$ is different from other $K(j)'s$, since by the nature of the experiment, there is no self transition from the x to itself.

Test for Transition Probabilities across Contexts:

To test the randomness of the syllable generation mechanism, it is then natural to test whether knowing the previous syllable in anyway changes the probability of observing the next syllable. In other words, we would like to test if the probability of a mouse choosing the syllable k under a context c depends on the previously used syllable or the mouse is generating the syllable k simply according to its overall preference for k under the context c. Therefore, we would like to test if $\pi_{k|j(i,c)} \propto \pi_{k|(i,c)}$ for all $k \in K(j)$ for all i, c, where $\pi_{k|(i,c)}$ denotes the overall preference of the ith mouse to select syllable k under context c. Since $\sum_{k \in K} \pi_{k|(i,c)} = 1$ and $\sum_{k \in K(x)} \pi_{k|x(i,c)} = 1$, for $j \in \{d, m, s, u\}$ we would like to test if $\pi_{k|j(i,c)} = \pi_{k|(i,c)}$ for all $k \in K$. Similarly, when $j = x$, since self-transitions are no longer allowed, we would want to test if $\pi_{k|x(i,c)} = \pi_{k|(i,c)}(1 - \pi_{x|(i,c)})^{-1}$ for $k \in K(x) = \{d, m, s, u\}$.

	d	m	s	u	x
d	$\pi_{d\|d(i,c)}$	$\pi_{d\|m(i,c)}$	$\pi_{d\|s(i,c)}$	$\pi_{d\|u(i,c)}$	$\pi_{d\|x(i,c)}$
m	$\pi_{m\|d(i,c)}$	$\pi_{m\|m(i,c)}$	$\pi_{m\|s(i,c)}$	$\pi_{m\|u(i,c)}$	$\pi_{m\|x(i,c)}$
s	$\pi_{s\|d(i,c)}$	$\pi_{s\|m(i,c)}$	$\pi_{s\|s(i,c)}$	$\pi_{s\|u(i,c)}$	$\pi_{s\|x(i,c)}$
u	$\pi_{u\|d(i,c)}$	$\pi_{u\|m(i,c)}$	$\pi_{u\|s(i,c)}$	$\pi_{u\|u(i,c)}$	$\pi_{u\|x(i,c)}$
x	$\pi_{x\|d(i,c)}$	$\pi_{x\|m(i,c)}$	$\pi_{x\|s(i,c)}$	$\pi_{x\|u(i,c)}$	-
Total	1	1	1	1	1

Table 1: Table of transition probabilities.

	d	m	s	u	x
d	$\pi_{d\|(i,c)}$	$\pi_{d\|(i,c)}$	$\pi_{d\|(i,c)}$	$\pi_{d\|(i,c)}$	$\pi_{d\|(i,c)}/\{1 - \pi_{x\|(i,c)}\}$
m	$\pi_{m\|(i,c)}$	$\pi_{m\|(i,c)}$	$\pi_{m\|(i,c)}$	$\pi_{m\|(i,c)}$	$\pi_{m\|(i,c)}/\{1 - \pi_{x\|(i,c)}\}$
s	$\pi_{s\|(i,c)}$	$\pi_{s\|(i,c)}$	$\pi_{s\|(i,c)}$	$\pi_{s\|(i,c)}$	$\pi_{s\|(i,c)}/\{1 - \pi_{x\|(i,c)}\}$
u	$\pi_{u\|(i,c)}$	$\pi_{u\|(i,c)}$	$\pi_{u\|(i,c)}$	$\pi_{u\|(i,c)}$	$\pi_{u\|(i,c)}/\{1 - \pi_{x\|(i,c)}\}$
x	$\pi_{x\|(i,c)}$	$\pi_{x\|(i,c)}$	$\pi_{x\|(i,c)}$	$\pi_{x\|(i,c)}$	-
Total	1	1	1	1	1

Table 2: Null hypothesis to test.

The null hypothesis of interest is $H_0 : \pi_{k|j(i,c)} \propto \pi_{k|(i,c)}$ for all $j \in K, k \in K(j)$ for all $i = 1, \ldots, n$ and all $c \in C$. A combined Pearson's chi-squared test statistic for testing H_0 is

given by

$$T_{\chi^2} = \sum_{i=1}^{n} \sum_{c \in C} \sum_{k \in K} 1\{\widehat{\pi}_{k|(i,c)} > 0\} \frac{(n^1_{k(i,c)} - \widehat{\pi}_{k|(i,c)})^2}{\widehat{\pi}_{k|(i,c)}} + \sum_{i=1}^{n} \sum_{c \in C} \sum_{j \in K} \sum_{k \in K(j)} 1\{n_{j(i,c)} > 0\} \frac{(n_{k|j(i,c)} - n_{j(i,c)}\widehat{\pi}_{k|j(i,c)})^2}{n_{j(i,c)}\widehat{\pi}_{k|j(i,c)}}.$$

where $\widehat{\pi}_{k|(i,c)}$ and $\widehat{\pi}_{k|j(i,c)}$ are the mle of $\pi_{k|(i,c)}$ and $\pi_{k|j(i,c)}$, respectively, under H_0. For fixed i, c pair, the likelihood function is given by

$$L(\{\mathbf{Y}_{i,c}\}_{i=1}^{n}, \{\pi_{k|(i,c)}\}_{k \in K}, \{\pi_{k|j(i,c)}\}_{j \in K, k \in K(j)}) = \prod_{k \in K} \pi_{k|(i,c)}^{n^1_{k(i,c)}} \prod_{j \in K} \prod_{k \in K(j)} \pi_{k|j(i,c)}^{n_{k|j(i,c)}},$$

Under H_0, the likelihood reduces to

$$L(\{\mathbf{Y}_{i,c}\}_{i=1}^{n}, \{\pi_{k|(i,c)}\}_{k \in K}, \{\pi_{k|(i,c)}\}_{k \in K}) = \prod_{k \in K} \pi_{k|(i,c)}^{n^1_{k(i,c)}} \prod_{k \in K} \pi_{k|(i,c)}^{n_{k|(i,c)}} \{1 - \pi_{x|(i,c)}\}^{-n_{|x(i,c)}},$$

where $n_{k|(i,c)} = \sum_{j \in K} n_{k|j(i,c)}$ and $n_{|x(i,c)} = \sum_{k \in K(x)} n_{k|j(i,c)}$. The first part is again independent multinomial and so we have $\widehat{\pi}_{k|(i,c)} = n^1_{k(i,c)}$. To avoid the problem of estimation of the initial distribution parameters based on only a single observation, all the mice may be assumed to have the same initial distribution. Assumptions on these independent nuisance distributions do not affect the test of interest as in either case the contribution of the initial distributions to the test statistic vanishes and a combined test statistic is obtained as

$$T_{\chi^2} = \sum_{i=1}^{n} \sum_{c \in C} \sum_{j \in K} \sum_{k \in K(j)} 1\{n_{j(i,c)} > 0\} \frac{(n_{k|j(i,c)} - n_{j(i,c)}\widehat{\pi}_{k|j(i,c)})^2}{n_{j(i,c)}\widehat{\pi}_{k|j(i,c)}}.$$

Here we used the sum of the individual T_{χ^2} values for different (i, c) pairs to test the combined null hypothesis. Such tests are highly conservative, i.e., a significant p-value may be taken to indicate very strong evidence against the combined null hypothesis. The mle's $\widehat{\pi}_{k|j(i,c)}$ are functions of $\widehat{\pi}_{k|(i,c)}$, the mle's of $\pi_{k|(i,c)}$, which do not have closed form analytical expressions and thus have to be estimated using numerical optimization methods. The difference in the number of free parameters between the alternative and the null for each i, c pair is $df(i, c) = \sum_{j \in K}(|K(j)| - 1) - (|K| - 1)$. As $T_{i,c} \to \infty$, we have that $T_{\chi^2} \to \chi^2_{df}$ with $df = \sum_{i=1}^{n} \sum_{c \in C} df(i, c)$.

For the mice data set, we have $T_{\chi^2}(obs) = 12811.28$ with $df = 720$ and p-value < 0.0001. Out of the 48 tests for different (i, c) pairs, 33 produced p-values less than the conservative Bonferroni level 0.05/48. These results provide strong statistical evidence that knowing the

current syllable significantly influences the probability of occurrence of the next syllable.

Test for Differences in Contexts across Syllables:

The null hypothesis of interest is $H_0 : \pi_{k|j(i,c)} = \pi_{k|j(i,c')} = \pi_{k|j(i)}$, say, for all $c, c' \in C$. A combined Pearson's Chi-Squared test statistic for testing H_0 is given by

$$T_{\chi^2} = \sum_{i=1}^{n} \sum_{j \in K} \sum_{c \in C} \sum_{k \in K(j)} 1\{n_{j(i,c)} > 0\} \frac{(n_{k|j(i,c)} - n_{j(i,c)}\widehat{\pi}_{k|j(i)})^2}{n_{j(i,c)}\widehat{\pi}_{k|j(i)}},$$

where $n_{j(i,c)} = \sum_{k \in K(j)} n_{k|j(i,c)}$ and $\widehat{\pi}_{k|j(i)}$ is the mle of $\pi_{k|j(i)}$ under H_0. For a fixed i, j pair, the likelihood function is given by the multinomial probability law

$$L(\{\mathbf{Y}_{i,c}\}_{i=1,s=1}^{n,S}, \{\pi_{k|(i,c)}\}_{c \in C, k \in K}, \{\pi_{k|j(i,c)}\}_{c \in C, k \in K(j)}) = \prod_{c \in C} \left\{ \prod_{k \in K(j)} \pi_{k|j(i,c)}^{n_{k|j(i,c)}} \right\}, \qquad (1)$$

Under H_0, $\widehat{\pi}_{k|j(i)}$ are thus given by $\widehat{\pi}_{k|j(i)} = \sum_{c \in C} n_{k|j(i,c)} / \sum_{k \in K(j)} \sum_{c \in C} n_{k|j(i,c)}$. The number of parameters in (1) under H_0 is $\left|\{\pi_{k|j(i)}\}_{k \in K(j)}\right| = \{|K(j)| - 1\}$, whereas the number of parameters under the alternative model is $\left|\{\pi_{k|j(i,c)}\}_{c \in C, k \in K}\right| = C\{|K(j)| - 1\}$. The difference is given by $df\{j(i)\} = \{K(j) - 1\}(C - 1)$. Therefore, we have $T_{\chi^2} \to \chi^2_{df}$ with $df = \sum_{i=1}^{n} \sum_{j \in K} df\{j(i)\}$ as $n_{j(i,c)} \to \infty$.

For the mice data set, we obtained $T_{\chi^2}(obs) = 5167.109$ with $df = 684$ and p-value < 0.0001, indicating that the syllable sequences vary significantly across different contexts. We next performed pairwise comparisons using combined Chi-Squared tests for each pair of contexts. The results are presented in Table 3. The p-values for all pairwise comparisons equal zero, providing strong statistical evidence that all the contexts are also pairwise significantly different.

	UR	FE	AF	AM
UR	-	2293.397	2018.810	1238.883
FE	-	-	875.380	1401.499
AF	-	-	-	614.941
AM	-	-	-	-

Table 3: The values in cell (c, c') represent the observed value of the Chi-squared test statistic for pairwise comparison between contexts c and c'. All the tests had dfs 228 and all p-values were < 0.0001.
